# ICARION: A Modular Framework for Ion Trajectory Simulation in Electric Fields and Neutral Gas Environments

**DOI:** 10.1021/jasms.6c00235

**Published:** 2026-09-03

**Authors:** Christoph Schaefer, Juraj Jašík, Patrik Španěl

**Affiliations:** 86875 Heyrovský Institute of the Czech Academy of Sciences, Dolejškova 3, Prague 8 18223, Czech Republic

**Keywords:** trajectory simulation, ion mobility, reactions, collisions, computational chemistry

## Abstract

Ion transport in electric fields and neutral gases underlies the operation of ion mobility spectrometers, mass spectrometers, and coupled IMS-MS instruments. Numerical modeling of such systems requires consistent treatment of field-driven motion, stochastic ion-neutral collisions, and reactions across different pressure and field regimes. Many IMS-MS instruments operate in transitional regimes, requiring explicit particle-based treatment of collisions. Here, we present ICARION (Ion Collision And Reaction IntegratiON), an open-source, modular, particle-based simulation framework for reproducible simulations of ion transport, collisions, and reactions. ICARION is intended for mechanistic studies of ion dynamics, exploration of new concepts in IMS, MS, and related techniques, and rapid and reproducible screening of operating parameters and geometries for instrument development and optimization. Its modular architecture combines various selectable field, collision, and reaction models with structured configuration and data processing. Multiple instrument domains with different electric fields, geometries, and environmental conditions can be seamlessly combined in a single simulation of coupled IMS-MS instruments. This work validates ICARION rather than introducing new physical models. Validation includes collisional thermalization, bimolecular reaction kinetics, ion transport in homogeneous electric fields validated with established tools, and coupled multidomain simulations. Across these cases, ICARION reproduces the expected behavior of the selected physical models and enables continuous simulations across domains differing by orders of magnitude in pressure and field time scales. While externally generated field maps can be integrated as needed, a planned internal field solver will enable the calculation of electric fields directly from the user-defined electrode configurations. The modular architecture also facilitates the integration of additional physical models.

## Graphical abstract

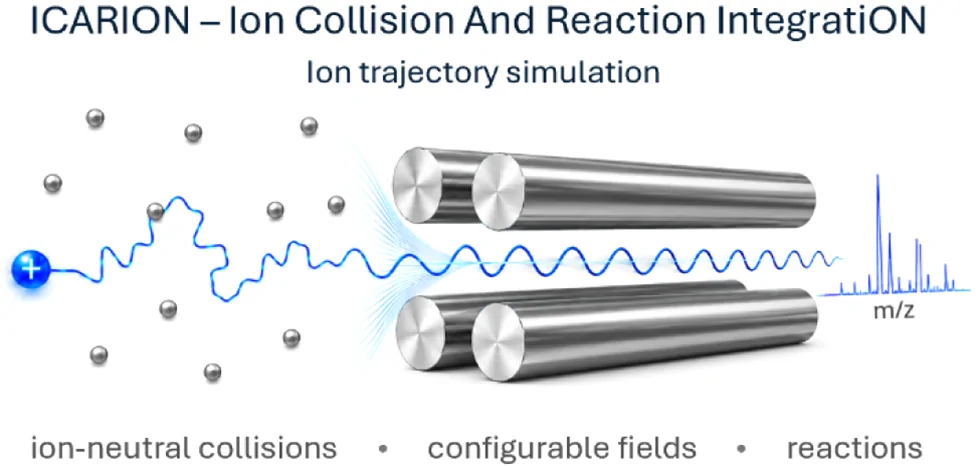

## Introduction

Ion transport in electric fields and neutral gases underlies the operation of a wide range of analytical instruments, including ion mobility spectrometers (IMS), radio-frequency (RF) ion traps, and hybrid IMS-MS systems.^1,2^ Depending on pressure and electric field strength, ion motion spans distinct transport regimes. At very low pressures, where the mean free path is much greater than the characteristic electrode dimensions, ion trajectories are nearly collision-free and deterministic. At high pressures, frequent collisions lead to diffusive transport that can be described statistically in terms of ensemble-averaged drift and diffusion.^3–5^ In contrast, many modern IMS-MS platforms operate in an intermediate-pressure regime, where the ion mean free path becomes comparable to the electrode dimensions. In this regime, individual ion-neutral collisions must be treated explicitly using stochastic, particle-based Monte Carlo approaches,^6,7^ as effective drag forces or purely statistical descriptions no longer accurately capture the underlying physics.

Modern mass spectrometers typically consist of multiple instrument regions, such as drift cells, ion guides, and RF trapping or filtering devices. In numerical simulations, these regions can be treated as separate instrument domains, each characterized by its own environmental conditions (pressure, temperature, and gas composition) and electric field configuration. Ions therefore traverse a sequence of domains that may differ strongly in their physical conditions, ranging from highly collisional drift cells to nearly collision-free trapping or *m*/*z* analysis stages. Accurate modeling of such instruments requires simulation approaches that consistently describe the ion motion across heterogeneous domains.

In low-field IMS, the collision cross section (CCS) obtained from the measured mobility using the fundamental equation of ion mobility corresponds to the first-order collision integral Ω^(1,1)^.^4^ This collision integral is obtained by integrating the momentum transfer efficiency induced by collisions over the impact parameters and averaging over the relative orientations of ions and neutrals to obtain the collision-energy-dependent momentum transfer cross section, followed by a transport-weighted averaging over the distribution of ion-neutral relative speeds. Therefore, the CCS is a transport quantity that depends on the neutral gas, the ion-neutral interaction potential, and the temperature, and is not a purely geometric area.

Numerical ion trajectory simulation is therefore an essential tool for instrument development, exploration of new concepts in MS, and data interpretation,^8,9^ enabling quantitative prediction of ion mobilities, transmission efficiencies, and reaction kinetics under experimentally relevant conditions.^10–14^ While statistical and analytical transport models provide valuable insights into equilibrated ion transport conditions, realistic instrument geometries and intermediate-pressure transport conditions—where mean free paths are comparable to electrode dimensions—require particle-based simulations that explicitly resolve both ion-neutral collisions and electric fields.^15–18^

Established simulation environments address these requirements from a complementary perspective. General-purpose ion-optics tools such as SIMION^6^ provide highly accurate electrostatic field calculations and deterministic trajectory propagation and are widely regarded as a reference standard for ion-optical simulations. Collisional processes can be incorporated through dedicated collision models and user-defined extensions; however, these capabilities are typically configured at the level of individual simulation setups and are not embedded in a unified, reproducible workflow that explicitly targets coupled transport-reaction problems across heterogeneous pressure regimes.

Particle-based drift simulation frameworks such as IDSimF^7,19^ or ITSIM^20^ focus on stochastic ion transport and reaction kinetics and provide explicit particle-based descriptions of ion-neutral collisions. In principle, IDSimF can be extended to simulate complex or multidomain instrument configurations. In practice, however, such coupled simulations require substantial custom code development, as multidomain behavior cannot be expressed solely through declarative configuration but must be implemented explicitly by the user.

Recently, IMoS 2 ^21^ has further advanced microscopic transport calculations by propagating ions through a local neutral gas environment and explicitly evaluating ion-neutral interactions from interaction potentials. This approach enables a detailed description of transport properties, field-dependent mobility, rotational dynamics, and ion heating, and can explicitly account for interactions with multiple gas particles simultaneously. However, its primary purpose is the extraction of transport properties rather than the full instrument-level simulation of ion trajectories across complete instrument domains or IMS-MS architectures with explicit geometries, boundary conditions, ion losses, and domain transitions.

As a result, simulations of coupled IMS-MS systems spanning drift, trapping, and *m*/*z* analysis domains often rely on bespoke code extensions, tool chaining, or simplifying assumptions, which complicate reproducibility and systematic validation across transport regimes.

Ion Collision And Reaction IntegratiON^22^ (ICARION) was developed for three complementary applications: mechanistic studies of ion motion in electric fields under the influence of collisions and reactions, the exploration of new concepts in IMS, MS, and related techniques, and rapid, reproducible screening of operating parameters and geometries for instrument development and optimization. It combines deterministic ion motion in electric fields with discrete collision and reaction events and supports continuous propagation across multiple instrument domains with different geometries, fields, and gas environments without requiring a manual transfer or reinitialization of the ion ensemble.

Its modular architecture allows for the selection of alternative physical descriptions (e.g., different collision models) and facilitates the integration of new models through stable interfaces. The current implementation offers analytical field descriptions and various selectable collision and reaction models, which are sufficient for many mechanistic simulations and rapid screening (see Section S3). Field maps and transport parameters generated by external tools can additionally be incorporated, e.g., when realistic, nonideal electric fields are required. The same interfaces also facilitate the integration of an internal numerical field solver, which is currently under development, to calculate electric fields from user-defined electrode geometries in future versions of ICARION without requiring restructuring of the instrument definitions, simulation engine, or data workflow.

The source code is available via GitHub (see Software Availability Statement), and the archived software release is available on Zenodo.^22^ Prebuilt executable packages for Windows and Linux are provided via the GitHub repository, allowing users to run ICARION without compilation.

The present work does not aim to introduce new ion-transport or reaction models. Instead, it focuses on the systematic validation of this unified, particle-based simulation framework that combines deterministic ion optics, stochastic ion-neutral collisions, and chemical reactions within a single reproducible numerical architecture. Emphasis is placed on transport regimes relevant to modern IMS-MS instrumentation, where ions traverse multiple instrument domains characterized by distinct pressures, field configurations, and time scales. The framework is validated using theoretical reaction kinetics and statistical speed distributions at equilibrium, for which independent reference solutions are available. Ion transport in a drift region with a uniform field is compared with the established ion trajectory simulation tools IDSimF and SIMION using comparable collision models that apply the same collision physics and match in terms of the input parameters and operating conditions.

Design philosophy, execution workflow, and configuration concepts are summarized in the next sections to provide context, while detailed implementation aspects are documented in the SI. This article therefore provides a validated and extensible framework for particle-based trajectory simulations of ion transport and chemistry in IMS-MS systems, together with openly available software and benchmark data sets to support its use and further development by the IMS-MS community.

## Design Philosophy and Development Goals

### Design Goals

ICARION was designed as a universal particle-based simulation framework for coupled ion transport and chemical reactions across heterogeneous IMS-MS environments.

ICARION is not primarily intended as a standalone tool for predicting transport parameters such as ion mobility or collision cross sections. Rather, the implemented collision models serve to simulate ion trajectories and thus propagate ion ensembles at the instrument level in the presence of electric fields and gas environments. The required level of physical detail, particularly in the collision model and in the description of the electric field, depends on the intended application. Computationally efficient, physically justified approximations are useful for rapid screening and simulations of large ion ensembles, whereas more detailed collision models based on explicit ion-neutral interaction potentials are required when ion transport depends strongly on collision energy or specific ion-neutral interactions. Likewise, field maps become relevant when nonideal electric fields must be taken into account.

A central design principle of ICARION is the modularity of physical models and numerical components. Field descriptions, collision models, reaction kinetics, force contributions, and numerical integrators are treated as interchangeable modules that can be combined consistently without modification of the core simulation engine. This approach enables the use and comparison of alternative physical models under identical numerical conditions and facilitates the implementation of new physical models through the use of stable interfaces. If necessary, externally generated field maps and transport parameters can be used as inputs, while a future internal numerical field solver can be integrated via the same interfaces.

The framework was evaluated with respect to numerical consistency, transparency, and reproducibility. The predictive accuracy of a given simulation depends on the assumptions made, the parametrization, the scope of applicability of the selected physical models, and the numerical settings, and must therefore be evaluated with regard to the intended application. To meet reproducibility and transparency as central design goals, all simulation parameters are either defined directly in structured configuration files or are derived from them. Defaults are clearly specified and documented in accordance with a strict single-source-of-truth (SSOT) philosophy, while all outputs and metadata are recorded in a standardized, self-describing format. This ensures that simulation results can be reproduced deterministically and independently of the execution environment while maintaining full transparency of the physical and numerical assumptions. In practice, this is implemented via declarative JSON-based input configurations and standardized HDF5 ^23^ output files, which together encode the parameters and metadata required for reproducible simulations.

### Execution Workflow

The general execution workflow of an ICARION simulation is shown in Figure 1. The SimulationEngine module acts as the central coordinator for all simulation components. Consistent with a strict separation of concerns, it does not encode any instrument- or model-specific logic but orchestrates the execution of independently defined physical and numerical modules. All simulation settings are initialized from the validated configuration prior to execution, including descriptions of the domains, physical models, numerical integrators, and output handlers.

**Figure 1:**
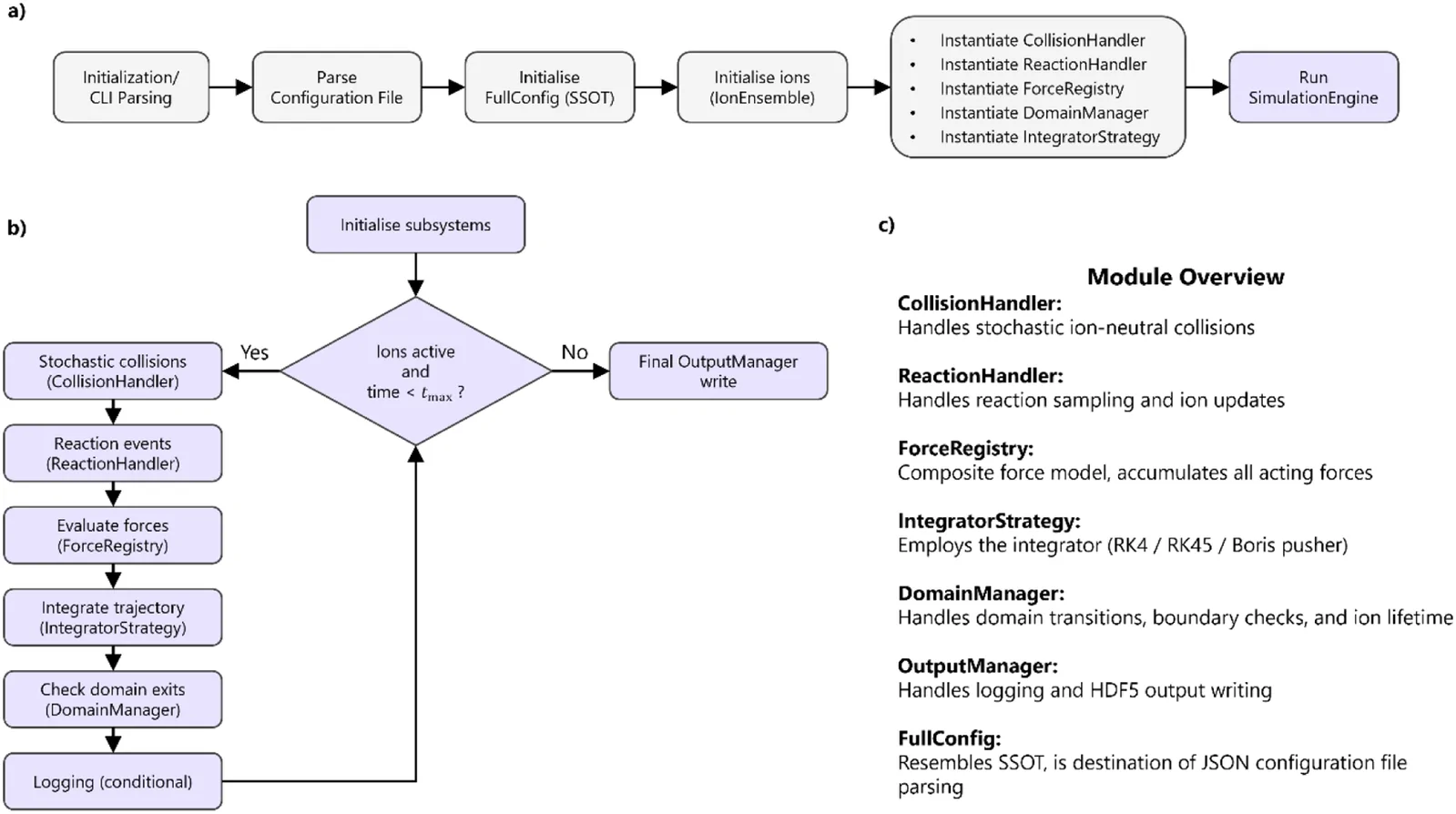
(a) High-level workflow of a complete ICARION simulation run, including configuration parsing, subsystem initialization, and invocation of the SimulationEngine. (b) Detailed loop executed by the SimulationEngine. In each iteration, the engine resolves the active domain, applies stochastic collisions and reaction events, evaluates forces, integrates the ion trajectory, and performs boundary actions and conditional logging. The loop terminates when no active ions remain or when the maximum simulation time is reached. (c) Overview of the physics and control modules initiated by each simulation run.

During execution, the simulation advances in discrete time steps. At each step, active ions are processed independently: optional space-charge contributions are updated according to the selected model, stochastic collision and reaction events are sampled, and forces from electric and magnetic fields are evaluated. Collisional effects are treated either explicitly via stochastic sampling, particularly for intermediate pressures, or implicitly via an ensemble-averaged drag force, particularly for high-pressure simulations, depending on the selected collision model. Subsequently, ion trajectories are propagated by using the selected numerical integrator. Boundary conditions and domain transitions are enforced after each propagation step. Output is buffered and written at user-defined intervals to ensure consistent temporal sampling while minimizing I/O overhead. The simulation terminates when either the maximum simulation time is reached or no active ions remain, after which the final output and metadata are written.

## Materials and Methods

All simulations were performed using the ICARION simulation framework (v1.0.2 production code).^22^ The exact simulation commit (40210bfe) is recorded in the output metadata; no production code relevant to the simulations was changed between this commit and the final v1.0.2 release (0f5dfce3). The code was compiled against the required libraries (HDF5 1.10.10 and JSON libraries (jsoncpp 1.9.5 and nlohmann_json 3.11.3)), the GNU Compiler Collection (GCC) 13.3.0, and executed on a workstation running Windows Subsystem for Linux (WSL). Parallel execution employed OpenMP for shared-memory multithreading. No GPU backends were used in this study. The exact Git commit, the configuration files, the random number generator (RNG) seed values, and relevant build information are recorded in the output data, which is available in the associated data set stored in the National Data Repository.^24^

Simulation setups were fully defined in structured JSON configuration files that specified domain geometries, neutral gas properties, electric field parameters, numerical integration settings, and the selected physical models. All configuration files, raw simulation outputs, postprocessing scripts, and auxiliary input files required to reproduce the validation cases are also provided in the associated data set.

All simulations presented in this work use the built-in analytical models for the instrument-specific idealized electric fields. Consequently, no externally generated electric field maps are required to reproduce the simulations presented here. For the HSS simulations, the externally generated transport data consist of the CCS values derived from MobCal-MPI 2.0,^25^ as explained below. The GR-HSS simulations also use the molecular geometries obtained from the quantum chemical calculations described below.

### Ion Species and Structural Data

All simulations were conducted for three representative small ions commonly encountered in gas-phase ion–molecule reaction and ion mobility studies: hydronium (H_3_O^+^), protonated pentanal, and protonated 2,6-di-*tert*-butylpyridine (2,6-DTBP). These species span a modest but relevant range of *m*/*z* and CCS typical for volatile compounds (see Table 1), without extending into the macromolecular regime. They were selected as representative test cases rather than as a limitation of the framework: ICARION can also be applied to the ion transport of larger molecular or macromolecular ions, provided that the corresponding species parameters are defined. All species metadata were specified through the centralized Species Database JSON file.

**Table 1:** Simulated Protonated Ions, Nominal *m*/*z* Values, Charge States, and CCS^a^

Ion	Nominal *m*/*z*	Charge state *z*	CCS (Å^2^)
H_3_O^+^	19	1	24.7
Pprotonated pentanal	87	1	53.9
Pprotonated 2,6-DTBP	192	1	85.0

a The listed CCS values were calculated using the MobCal-MPI 2.0 software package^25^ in helium at 298 K.

### Applied Hard-Sphere Collision Models

The two validation cases that focus on thermalization from collisions and ion mobility transport use two stochastic elastic hard-sphere collision models: an effective, isotropic single hard-sphere scattering model (HSS) and a geometry-resolved, single-contact hard-sphere scattering model (GR-HSS), as schematically shown in Figure 2. A detailed explanation of both models is given in the SI (Section S3). Briefly, both models sample collision events from a Poisson process. In the following, *σ*_event_ denotes the collision event cross section, which is used in each time step to calculate the instantaneous collision rate according to $k_{\mathrm{coll}}=N\sigma_{\mathrm{event}}\left|{\mathrm{v⃗}}_{\mathrm{rel}}\right|$ , where *N* is the neutral number density and $\left|{\mathrm{v⃗}}_{\mathrm{rel}}\right|$ is the sampled ion-neutral relative speed. The Cartesian velocity components of neutral collision partners are drawn from the corresponding thermal normal distributions, resulting in a Maxwellian velocity distribution for the neutral ensemble, as detailed in the SI (Section S3).

**Figure 2:**
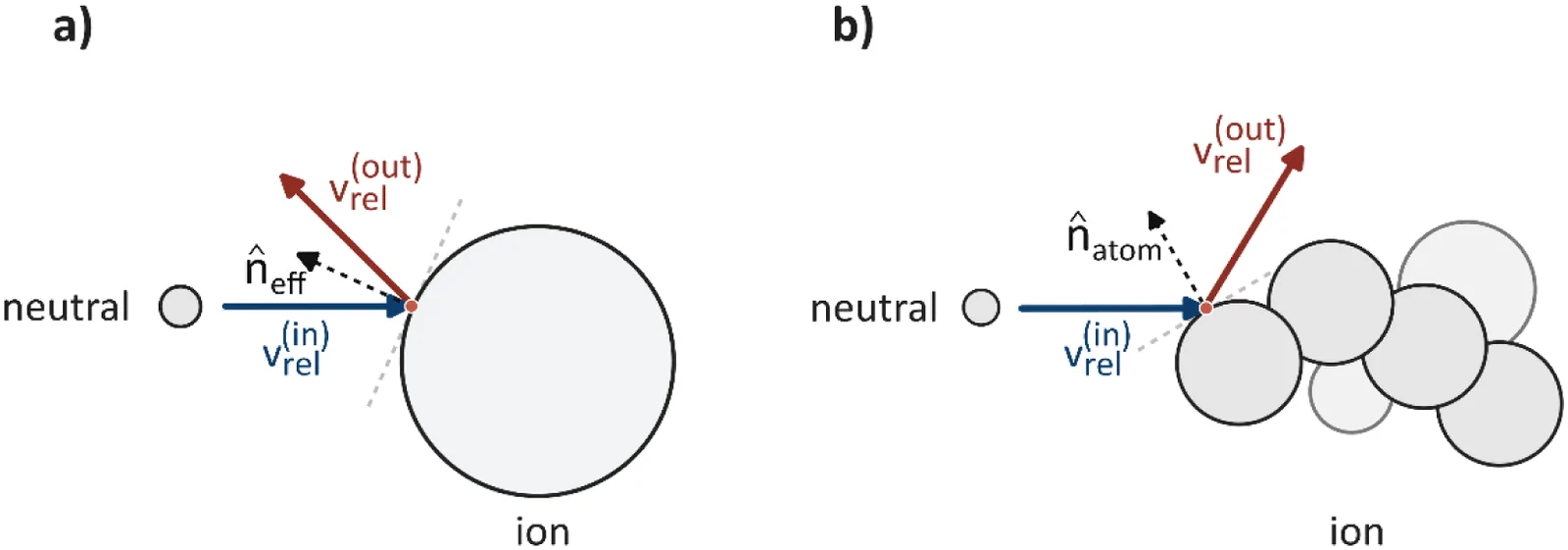
Schematic comparison of representative collision events in the HSS and GR-HSS models. (a) In HSS, the ion is represented by an effective single hard sphere, and the relative velocity direction after the collision is sampled isotropically in the center-of-mass reference frame; the illustrated surface normal n̂_eff_ is therefore an equivalent geometrical visualization of one sampled scattering event, not an explicitly evaluated collision normal. (b) In GR-HSS, the ion is represented by an oriented atom-centered hard-sphere geometry, and the local surface normal n̂_atom_ is obtained from the first ray-sphere intersection. In both cases, the collision is treated in the center-of-mass reference frame, and the magnitude of the relative velocity is conserved. Note that the representation is two-dimensional only for visualization. All calculations are fully three-dimensional.

In the HSS model, a single value is used as *σ*_event_, and an accepted event is treated by sampling an isotropic relative velocity direction after the collision in the center-of-mass reference frame, which is consistent with other Monte Carlo methods for hard spheres.^26^ The model requires neither an explicit ion geometry nor an ion-neutral interaction potential and therefore provides a computationally efficient approximation for rapid screening and large ion ensembles. In the GR-HSS model, *σ*_event_ is determined from the projected area of an ion orientation selected at random from a precomputed set of three-dimensional orientations, which is consistent with established geometry-based approaches using molecular projected areas.^27–29^ If an event is accepted, the same orientation is used to identify the first contact between the neutral and the atom-centered hard-sphere representations of the ion, using a consistent set of van der Waals radii from Mantina et al.,^30^ followed by a single elastic specular reflection at the local three-dimensional contact normal. Secondary contacts and multiple scattering, as in the exact hard-sphere scattering (EHSS) model,^31^ are not included.

Both HSS and GR-HSS treat collisions as specular and elastic and conserve the total translational kinetic energy of the ion-neutral pair. Diffuse re-emission, thermal accommodation, and transfer of translational energy to rotational or vibrational degrees of freedom are not taken into account. Another collision model not implemented in this work, diffuse hard-sphere scattering (DHSS),^32,33^ represents a different physical description in which nonspecular re-emission and energy accommodation can be incorporated. Such processes are not included in the HSS and GR-HSS implementations considered here.

The scope of the present work is a fundamental validation of the ICARION v1.0.x framework. Stochastic reaction sampling and thermalization from collisions are evaluated on the basis of theoretical reaction kinetics and statistical velocity distributions at equilibrium, respectively. Ion mobility transport and computational performance are compared with the established ion trajectory simulation tools IDSimF and SIMION, both of which implement a comparable effective single-hard-sphere approximation to ensure consistent collision physics under matched input parameters and operating conditions.

ICARION v1.1.0 introduced a collision model based on explicit ion-neutral interaction potentials to account for the dependence of elastic scattering on collision energy. However, its application and physical validation are beyond the scope of this work. Inelastic collisions are currently not included in any of the implemented collision models, and their implementation would require a separate model extension. The modular ICARION architecture is designed to enable such extensions without modifying the surrounding instrument, field description, or the remaining data handling framework.

### Quantum Chemical and Collision Cross Section Calculations

Three-dimensional molecular geometries and electrostatic partial charges for all three ions were obtained from density functional theory (DFT) with the ORCA 6.0 software package.^34^ Geometry optimizations and vibrational frequency calculations were conducted using the ωB97X-D3 functional^35^ with the def2-TZVPP basis set.^36^ The RIJCOSX approximation^37^ was employed for the evaluation of hybrid exchange, and all calculations used tight self-consistent field and geometry convergence criteria. CHELPG^38^ partial charges were extracted using a cubic grid with 0.1 Å spacing and a sampling radius of 3.0 Å. The resulting optimized molecular geometries were stored in the ICARION JSON geometry format and are included in the associated data set along with ORCA input and output files.

Optimized geometry and charge distributions were exported to MobCal-MPI 2.0,^25^ which was used to calculate the CCS under low-field conditions in helium at 298 K. The resulting CCS values were used as the collision event cross section *σ*_event_ in the HSS simulations. These values are determined using the trajectory method, in which the momentum transfer cross section is calculated as a function of ion-neutral relative speed, then averaged over all considered orientations, and finally transport-weighted averaging is performed over the relative speed distribution. Nevertheless, *σ*_event_ values used in both hard-sphere models remain independent of the instantaneous collision energy. The use of these constant values for Poisson sampling is part of the effective single-sphere approximation in the HSS model.

## Results and Discussion

The following section presents each validation case in increasing order of physical and instrumental complexity. Starting with fundamental microscopic benchmarks, such as collisional thermalization and reaction kinetics, we proceed to macroscopic transport validation in homogeneous electric fields, followed by a coupled IMS-quadrupole simulation to illustrate multidomain capability. A performance benchmark and its comparison to IDSimF and SIMION conclude this section.

Unless stated otherwise, helium was used as the neutral gas. For all simulations, the integration time step was chosen as Δ*t* ≈ *τ*_c_/50, where *τ*_c_ denotes the mean collision time derived from the local pressure *p*, background temperature *T*, collision cross section σ, and mean ion-neutral relative velocity *v̅*_rel_ according to (eq 1).

1 $$\tau_{c}=\frac{k_{B}T}{p\sigma {\mathrm{v̅}}_{\mathrm{rel}}}$$

This constraint ensures that the probability of multiple collision events occurring within a single simulation time step remains negligible and that the assumptions underlying stochastic sampling remain valid.

### Thermalization from Stochastic Collisions

The ability of the stochastic collision models implemented in ICARION to reproduce thermal equilibrium with a neutral gas was assessed under field-free conditions for H_3_O^+^, protonated pentanal, and protonated 2,6-DTBP.

Simulations were conducted over a pressure range from 0.2 to 2000 Pa in decadic steps and at background temperatures of 200, 300, 400, 700, and 1000 K. For each combination of ion species, pressures, and temperatures, 10,000 ions were initialized from an isotropic thermal velocity distribution at 0.1 K and propagated under field-free conditions without any gas flow. The simulation duration was chosen to correspond to 30, 100, and 140 mean collision times for H_3_O^+^, protonated pentanal, and protonated 2,6-DTBP, respectively, ensuring that steady-state thermal equilibrium was reached within the statistical uncertainty.

Both the effective single-sphere HSS model and the geometry-resolved GR-HSS model were evaluated. The purpose of this comparison is to verify whether the ions in both collision models reach an expected thermalized state. It is explicitly not intended to present either model as a general, correct microscopic description of the ion-neutral collision.

To quantify thermalization under the field-free conditions considered here, the instantaneous translational temperature of the ion ensemble was calculated from its mean kinetic energy as shown in (eq 2). Here, ⟨*υ* ^2^(t)⟩ denotes the ensemble-averaged squared ion speed at time *t* with ion mass *m*_ion_. Since neither an electric field nor bulk gas flow is present, the mean ensemble velocity remains close to zero, and *T*_ens_(*t*) describes the transient relaxation of the ion ensemble toward the ambient gas temperature. It should not be interpreted as the field-dependent effective temperature used in two-temperature theory.

2 $$T_{\mathrm{ens}}\left(t\right)=\frac{m_{\mathrm{ion}}}{3k_{B}}\langle v^{2}\left(t\right)\rangle$$

Figure 3(a) shows the normalized ensemble translational temperature *T*_ens_(*t*)/*T*_domain_ as a function of the scaled time *t*/*τ_c_* for the three ions at 300 K using the GR-HSS model, evaluated at 0.2 and 2000 Pa. For each ion, the low- and high-pressure curves overlap on this scale, demonstrating that equilibration dynamics are primarily governed by the number of ion-neutral collisions. The translational temperature converges to *T*_domain_ quickly within approximately 10–60 *τ_c_*, with heavier ions thermalizing more slowly because of their lower momentum-transfer efficiency in collisions.

**Figure 3:**
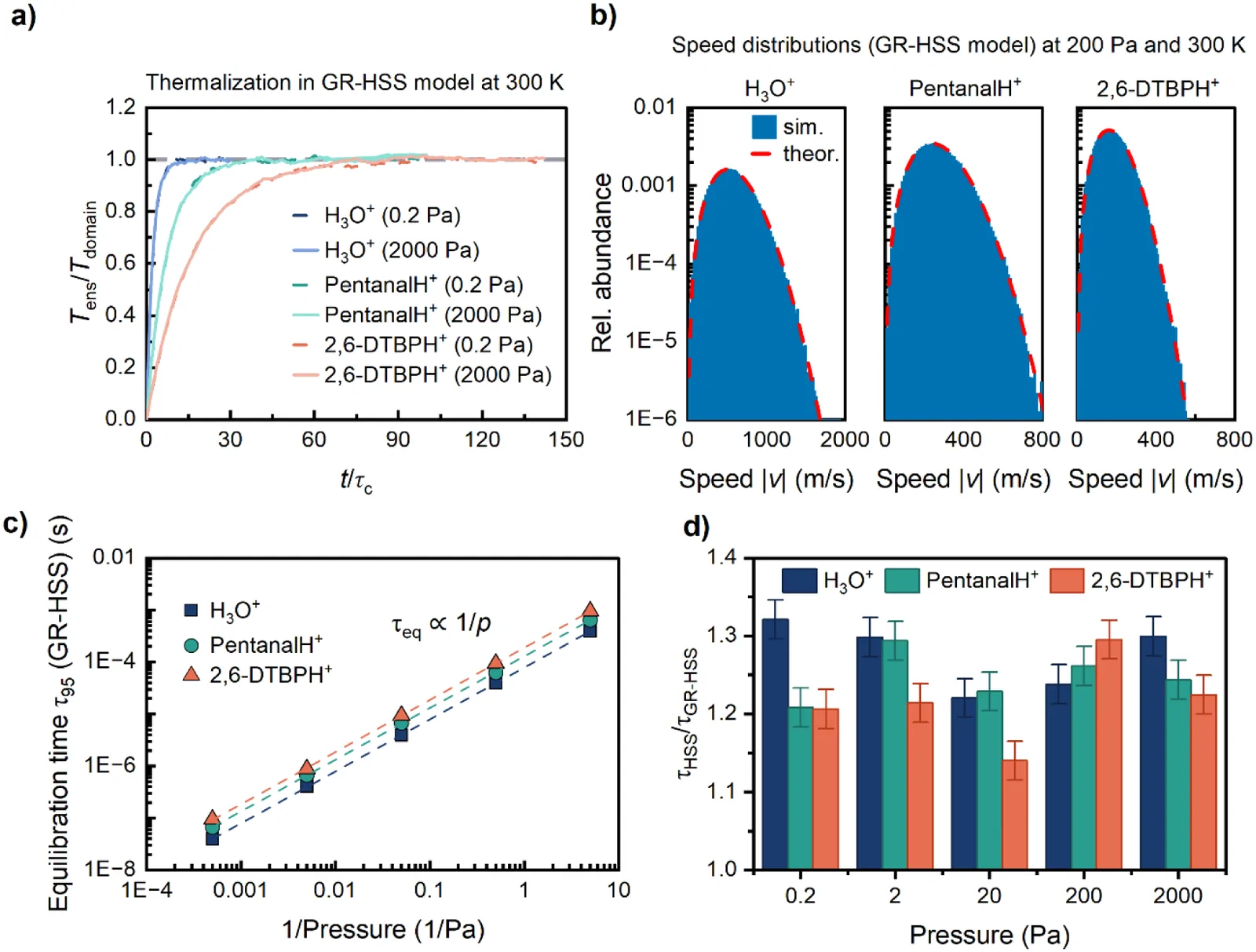
Thermalization under stochastic ion-neutral collisions. (a) Normalized ensemble translational temperature *T*_ens_/*T*_domain_ for all three ions at 300 K (GR-HSS model) for 0.2 and 2000 Pa. Low- and high-pressure curves overlap when plotted against *t*/*τ_c_*, confirming collision-governed equilibration. (b) Steady-state speed distributions at 200 Pa and 300 K. Simulated histograms agree with analytical Maxwell speed distributions over several orders of magnitude. (c) Equilibration time *τ*_95_ as a function of inverse pressure, showing the expected *τ*_95_ ∝ 1/*p* scaling. (d) Ratio of equilibration times predicted by the HSS and GR-HSS collision models. The error bars are not determined for each condition individually but are derived from the median standard deviation across 10 independent RNG seed values, repeated for selected data points, as explained in the Supporting Information (Section S5). Values close to unity would indicate similar equilibration dynamics, with HSS generally predicting slightly longer equilibration times.

Speed distributions obtained after equilibration are shown in Figure 3(b) for all three ions at 200 Pa and 300 K. The simulated distributions agree closely with analytical Maxwell speed distributions over several orders of magnitude in probability density, indicating that the stochastic collision models correctly reproduce not only the second moment but also the complete shape of the equilibrium speed distribution.

To further quantify equilibration dynamics, the equilibration time *τ*_95_, defined as the time required to reach *T*_ens_/*T*_domain_ = 0.95, was extracted for all conditions. Figure 3(c) shows that *τ*_95_ follows the expected scaling *τ*_95_ ∝ 1/*p* over four decades in pressure for all ions, consistent with kinetic expectations for hard-sphere-dominated scattering.

Equilibration times obtained with the HSS and GR-HSS collision models are closely correlated and of the same order of magnitude across the full pressure–temperature grid (Figure 3(d)). In the present benchmark, GR-HSS yields systematically shorter equilibration times than HSS. The equilibration times reported here are obtained from exponential fits to the translational temperature relaxation curves. Across all ions and conditions, *τ*_HSS_/*τ*_GR–HSS_ is typically in the range of approximately 1.14–1.32. Complete pressure–temperature grids and additional equilibration statistics (*τ*_95_, *τ*_99_, and the average number of collisions required for equilibration) are available in the associated data set.

This systematic offset between the two collision models is primarily explained by the different *σ*_event_ values used in Poisson sampling. In HSS, the MobCal-MPI output was used as the single *σ*_event_ value. In the geometry-resolved model, *σ*_event_ corresponds to the projected area of the sampled orientation (see “Applied Hard-Sphere Collision Models” and Section S3). The mean *σ*_event_ values in GR-HSS are approximately 1.24, 1.25, and 1.17 times larger for H_3_O^+^, protonated pentanal, and protonated 2,6-DTBP, respectively, than the corresponding *σ*_event_ values in HSS. The larger *σ*_event_ value in the GR-HSS model may seem counterintuitive, since the CCS derived from MobCal-MPI, which is used as *σ*_event_ in HSS simulations, includes contributions from long-range ion-neutral interactions, whose contribution to scattering generally decreases with increasing relative speed. However, the CCS from MobCal-MPI and the *σ*_event_ values derived from GR-HSS are not identical physical quantities and are derived from different scattering models. The former is a transport collision integral derived from an explicit ion-neutral interaction potential, while the latter is a geometric projected area. Another cause of the observed difference is the independent parametrization of the GR-HSS contact geometry. In ICARION, the element-specific atom-helium contact distances used to search for the first ion-neutral contact are determined by adding the respective van der Waals radii reported by Mantina et al.^30^ This provides a single, internally consistent literature-based set of parameters without ion-specific fitting to calculated or experimental ion mobility or CCS data. The resulting hard-sphere size therefore does not correspond to the finite repulsive interaction used in MobCal-MPI. The observed differences consequently reflect both the different physical quantities and the independent GR-HSS parametrization. Thus, the two collision models should not be interpreted as alternative predictions of the same CCS.

The different *σ*_event_ values nevertheless lead directly to different sampled collision event rates between HSS and GR-HSS and thus capture the trend and order of magnitude of the observed equilibration time ratios. Additional contributions may arise from stochastic sampling uncertainty and residual uncertainty in the relaxation fits, as supported by control simulations with different RNG seed values (see Section S5 in the SI for more details). Briefly, these control simulations yield standard deviations of approximately 0.01–0.04 across seed values for a given ion and fixed condition. The error bars in Figure 3(d) represent the median value of 0.025 of these calculated standard deviations to estimate the statistical uncertainty. These show that the differences in the equilibration time ratios between the various conditions can be largely explained by stochastic sampling uncertainty and the residual uncertainty in the relaxation fits. A mechanistic control simulation for protonated 2,6-DTBP, in which the *σ*_event_ in the HSS simulation was set to the mean GR-HSS value, reduced the ratio of equilibration times to 1.019 ± 0.015. This supports the interpretation that, under the conditions studied, the observed differences in equilibration times are dominated by differences in collision rates, with minor residual contributions from the different collision physics.

### Reaction Kinetics

To validate the stochastic sampling of ion–molecule reactions along ion trajectories, a bimolecular proton transfer from H_3_O^+^ to a neutral molecule M, forming MH^+^, was simulated under pseudo-first-order conditions. The purpose of this benchmark is to verify that the probabilistic reaction handling implemented in ICARION reproduces the expected temporal evolution of ion populations for well-defined reaction rates.

The reaction was treated as irreversible and bimolecular, with three fixed model rate coefficients of *k*_1_ = 1 × 10^–9^cm^3^/s, *k*_2_ = 2 × 10^–9^cm^3^/s, and *k*_3_ = 3 × 10^–9^cm^3^/s, representative of fast proton transfer reactions. Simulations were conducted at a background temperature of 300 K and a pressure of 400 Pa in helium, with the neutral M mixing ratio set to 1 ppm, thereby ensuring pseudo-first-order conditions with respect to the ion population.^39,40^

Reaction events were sampled stochastically by using a Poisson process. At each time step, the probability of a reaction occurring was computed from the reaction rate coefficient (see Section S3 for details). When a reaction event was accepted, the reacting ion species was updated through the centralized species database, including mass, charge state, collision cross section, and reduced ion mobility, before propagation continued.

Figure 4 shows the temporal evolution of the model H_3_O^+^ and MH^+^ ion populations for the three different reaction rate coefficients. In all cases, the decay of the reagent ion population follows an exponential behavior, while the product ion population increases correspondingly. The simulated ion counts are in excellent agreement with the analytical solution of the corresponding first-order rate equation, demonstrating correct probabilistic sampling of reaction events along ion trajectories. The final ion populations obtained at the end of the simulation are consistent with the expected reaction extent under the chosen conditions. For a reaction rate coefficient of 3 × 10^–9^ cm^3^/s, the simulated ion population yields approximately 70% MH^+^ product ions after 4 ms, which is in good quantitative agreement with the analytical prediction based on the effective pseudo-first-order rate coefficient.

**Figure 4:**
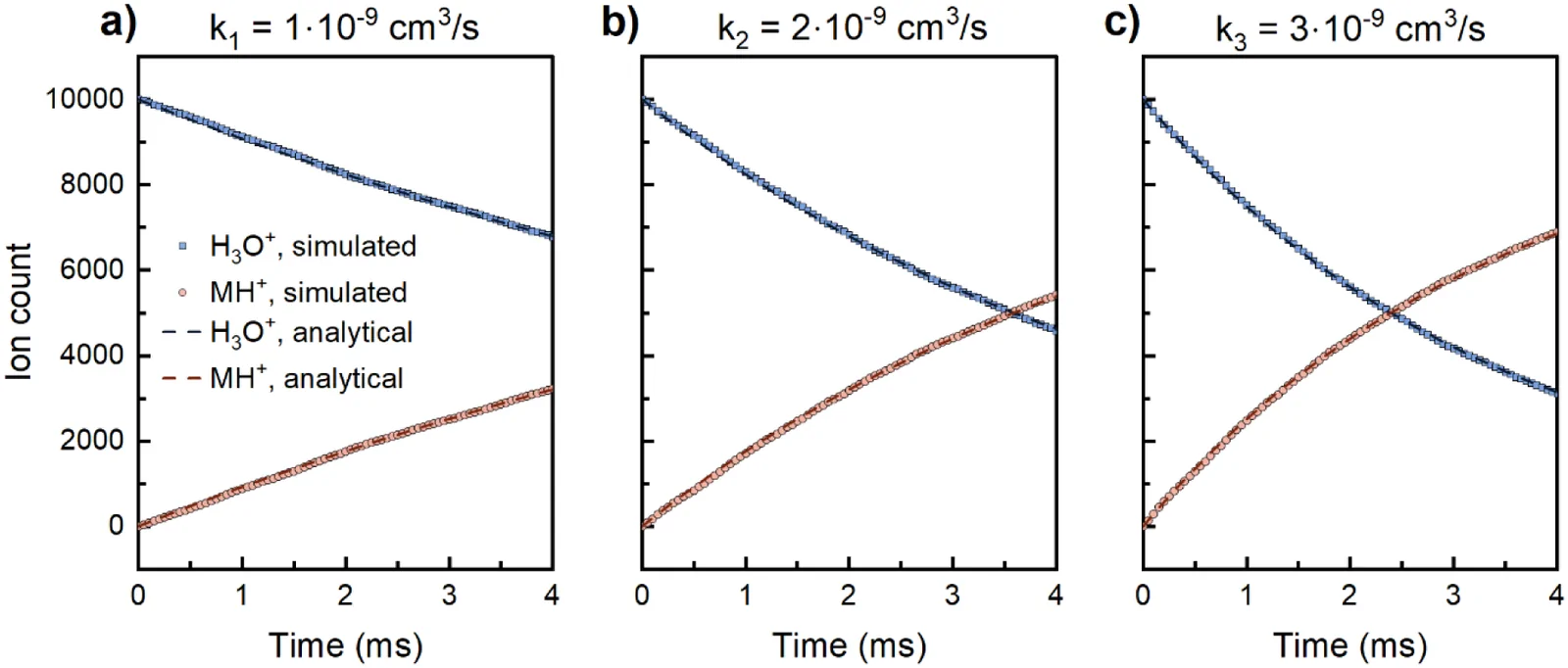
Validation of stochastic reaction sampling in ICARION. Temporal evolution of H_3_O^+^ and MH^+^ ion populations for a bimolecular proton transfer reaction under pseudo-first-order conditions at 300 K and 400 Pa with an M mixing ratio of 1 ppm. Markers indicate simulation results; dashed lines show analytical solutions for three different bimolecular reaction rate coefficients of (a) *k*_1_ = 1 × 10^–9^ cm^3^/s, (b) *k*_2_ = 2 × 10^–9^ cm^3^/s, and (c) *k*_3_ = 3 × 10^–9^ cm^3^/s.

These results confirm that the reaction handling in ICARION reproduces correct ensemble-averaged reaction kinetics for bimolecular ion–molecule reactions. This provides a validated basis for coupled transport-reaction simulations, which are known to affect arrival time distributions in ion mobility experiments.^41–43^

### Ion Transport in Drift Cells

In the next series of simulations, ion transport in uniform electric fields was investigated to validate stochastic particle-based ion mobility simulations and to benchmark ICARION against established trajectory simulation tools. Simulations were conducted in a cylindrical drift cell operated with helium as the neutral gas at 300 K. The drift cell had an effective length of 100 mm and an inner diameter of 10 mm. Pressures ranging from 20 to 2000 Pa were investigated, covering a regime in which stochastic ion-neutral collisions remain computationally feasible while spanning a broad range of collision frequencies. Reduced field strengths of 3, 5, 7, 8.5, and 10 Td were applied as representative IMS operating conditions in the range of low-to-moderate reduced field strengths.

Simulations were conducted for protonated pentanal in helium. Ions were initialized with thermal velocity distributions at 300 K and propagated through the drift cell until they exited the domain. Drift times were extracted from the simulated arrival time distributions and converted to reduced mobility.

Ion transport simulations were conducted using the two stochastic collision models implemented in ICARION and already employed in the thermalization validation case: the isotropic HSS model and the geometry-resolved GR-HSS model. For cross-code benchmarking, ICARION-HSS results were compared to isotropic single-sphere simulations conducted with IDSimF (HS) and with SIMION (HS1), widely regarded as a reference standard for ion trajectory simulations. In all isotropic single-sphere simulations, collision cross sections were matched to identical effective values to ensure a fair numerical comparison. Further details on the simulation configurations are provided in Section S4.

Figure 5(a) shows the reduced ion mobility as a function of pressure for representative *E*/*N* values of 3 and 10 Td. At moderate and higher pressures, particularly above 200 Pa, ICARION-HSS, IDSimF, and SIMION yield consistent ion mobilities across the investigated field range. Deviations between the three implementations typically remain below 1% under these equilibrated conditions, confirming numerical agreement of stochastic collision handling and trajectory propagation across the three independent simulation frameworks. The reduced ion mobilities of 10.10 to 10.32 cm^2^/(V s), determined from the simulated arrival times using the HSS model, agree well with the MobCal-MPI value of 10.17 cm^2^/(V s), which is expected since *σ*_event_ in the HSS model was parametrized based on the MobCal-MPI value. Nevertheless, this agreement further demonstrates the internal consistency of the HSS implementation and confirms that it reproduces the expected transport behavior for a given input value of *σ*_event_.

**Figure 5:**
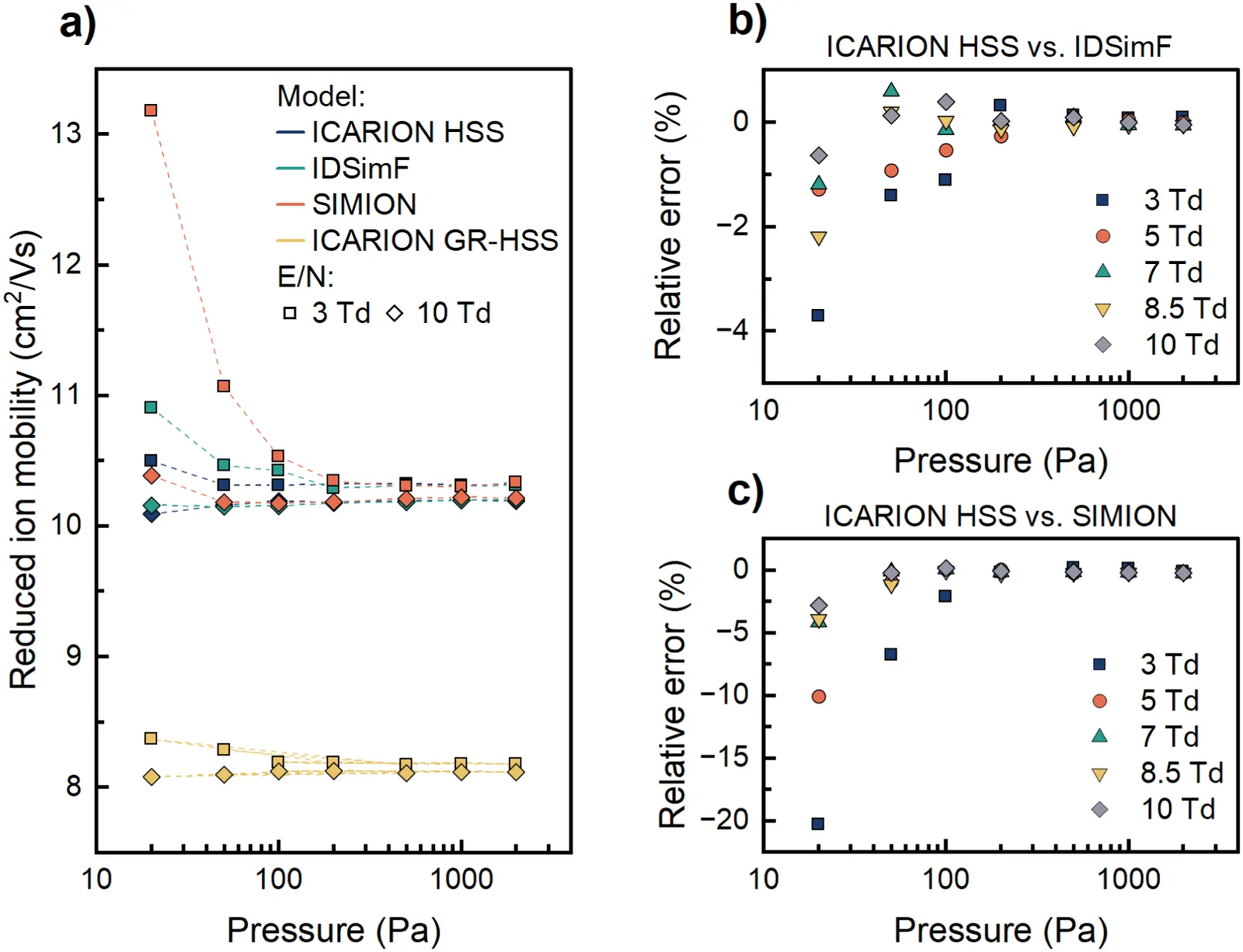
Reduced ion mobility of protonated pentanal in helium (300 K) calculated using ICARION, IDSimF, and SIMION across pressures and *E*/*N* values. (a) Reduced ion mobilities depending on pressure (20–2000 Pa) at 3 and 10 Td using ICARION (HSS and GR-HSS models), IDSimF (HS), and SIMION (HS1). (b) Relative error between ICARION-HSS and IDSimF across pressures and *E*/*N* (3–10 Td). (c) Relative error between ICARION-HSS and SIMION for the same conditions.

At lower pressures, systematic deviations in the outputs of the three tools became apparent. While SIMION in particular yields higher apparent mobilities, ICARION-HSS and IDSimF remain in closer agreement, as shown in Figure 5(b) and (c). These deviations reflect the transition toward an ion transport regime in which the actual axial velocity is not fully equilibrated over the entire finite drift time, rather than numerical inconsistencies between the simulation tools. Because of the low collision frequencies under these conditions, the equilibration time can become a substantial fraction of the observed drift time. The extracted arrival time, and thus the inferred apparent ion mobility, therefore retains sensitivity to the initial ion velocity distribution (see Section S6). The observed differences between the simulation tools can be attributed to small differences in the initial velocity distributions and the ion initialization conditions. These results demonstrate numerical agreement among ICARION, IDSimF, and SIMION under equilibrated conditions when comparable collision models and matched input parameters are used. The remaining deviations at low pressure reflect intrinsic nonequilibrium transport effects rather than differences in the underlying numerical implementations.

The GR-HSS model yields systematically lower reduced ion mobilities than the isotropic HSS model across the investigated pressures and reduced electric field strengths. The ion mobility ratio *K*_0,HSS_/*K*_0,GR–HSS_ is approximately 1.24–1.27, which agrees with both the ratio of equilibration times discussed above and the ratio of *σ*_event_. For protonated pentanal in helium, the mean value of *σ*_event_ in GR-HSS is 1.25 times larger than the corresponding HSS value. As in the validation case of thermalization from collisions, we performed control simulations to assess statistical uncertainty and the effect of using identical *σ*_event_ values (see Section S6). These results again support the interpretation that, under the conditions studied here, the observed difference in ion mobility is dominated by differences in *σ*_event_, with only a small residual contribution from the different collision physics. Thus, the lower ion mobility in the GR-HSS model can be attributed primarily to the higher collision event rate. We emphasize again that the larger *σ*_event_ determined with GR-HSS reflects the parametrization using the consistent data set of van der Waals radii from Mantina et al.,^30^ which, while it may limit predictive accuracy, avoids ion- or data-specific empirical fitting.

A complete data set of reduced ion mobilities for all settings and control simulations is available in the associated data set.

### Multidomain Simulation: IMS-Quadrupole Coupling

To validate ICARION’s ability to simulate ion transport across coupled instrument domains, a multidomain IMS-quadrupole configuration was simulated for H_3_O^+^, protonated pentanal, and protonated 2,6-DTBP. The multidomain setup consists of a drift cell (100 mm length, 10 mm radius) operated under collisional conditions (10 mbar helium, 298 K), followed by an RF quadrupole (100 mm length, 4 mm characteristic radius) operated as a mass filter at low pressure (10^–6^ mbar), as illustrated in Figure 6. This configuration represents a minimal yet representative IMS-MS coupling scenario spanning collisional and nearly collision-free transport regimes. From a practical standpoint, the coupled simulation is defined by adding only a second domain block to the configuration, while all physical models and numerical settings remain unchanged.

**Figure 6:**
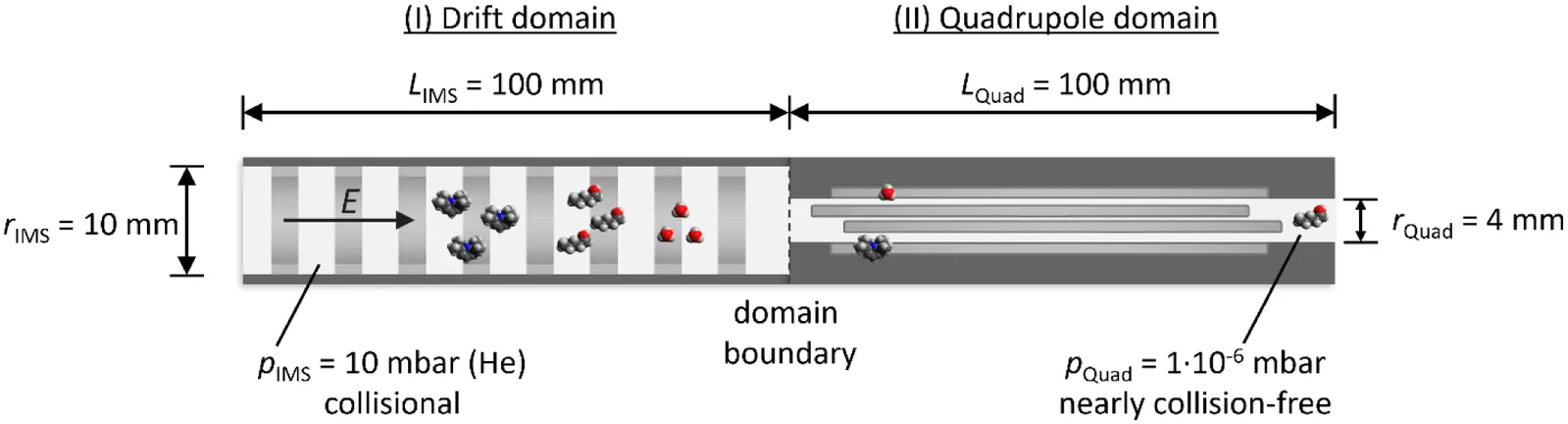
Schematic representation of the coupled multidomain IMS-quadrupole instrument. Ions are first separated in a drift cell (length 100 mm, radius 10 mm) operated in helium under collisional conditions (10 mbar) in a uniform electric field. Upon exiting the drift cell, ions enter the second domain, representing an RF quadrupole mass filter (length 100 mm, radius 4 mm), operated under nearly collision-free conditions (*p*_Quad_ = 1 × 10^–6^ mbar).

In the first domain, ions are separated according to their mobilities in helium under a uniform electric field. Upon exiting the drift cell, ions enter the second domain, which acts as a quadrupole mass filter, where ion motion is predominantly governed by time-dependent RF and DC electric fields. The two domains differ by 7 orders of magnitude in pressure and by the nature of the governing electric fields.

Figure 7(a) shows the arrival time distributions at the domain boundary for the three ion species, illustrating the ion mobility separation achieved prior to domain transition. The three ion packets reach the domain boundary at distinct mean arrival times of approximately 90, 171, and 251 μs for H_3_O^+^, protonated pentanal, and protonated 2,6-DTBP, respectively. Ions enter the quadrupole sequentially according to these residence times in the drift cell, resulting in temporally separated ion packets at the domain boundary.

**Figure 7:**
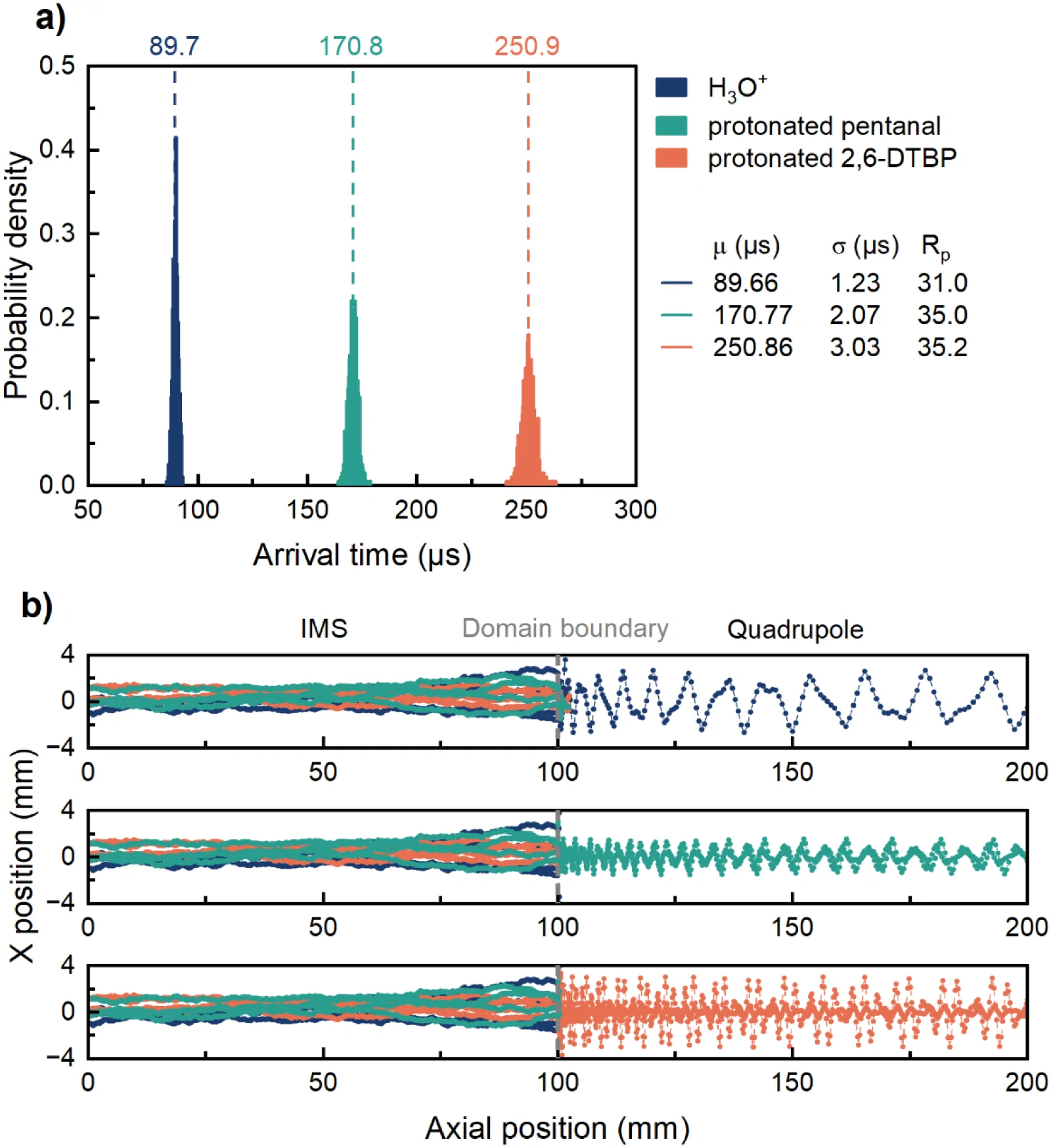
Multidomain simulation of an IMS-quadrupole instrument. (a) Arrival time distributions at the domain boundary for H_3_O^+^, protonated pentanal, and protonated 2,6-DTBP in helium, showing ion mobility separation before entry into the quadrupole domain. (b) Representative ion trajectories across the IMS and quadrupole mass filter domains from three simulations with different quadrupole settings, demonstrating continuous propagation of ions across the domain boundary. The quadrupole mass filter settings were configured to selectively transmit only one of the three ions (top: H_3_O^+^, center: protonated pentanal, bottom: protonated 2,6-DTBP). In each simulation, 1000 ions per species were simulated, but only four exemplary trajectories are shown for better visibility. To demonstrate reproducibility, the same RNG seed was used so that the trajectories in the drift cell agree across all three runs.

To illustrate the coupling of the two domains and the reproducibility of the particle-based simulations, three independent simulations were conducted with identical IMS conditions but different quadrupole mass filter settings, each tuned to selectively transmit only one of the three species. Figure 7(b) shows representative ion trajectories across both domains for these simulations. In the drift cell, trajectories exhibit diffusive motion superimposed on directed drift, characteristic of stochastic ion-neutral collisions. Upon entering the quadrupole domain, trajectories transition smoothly to an RF-driven oscillatory motion with negligible collisional effects.

For all of the simulations, the same RNG seed value was used. Therefore, the corresponding ion trajectories in the drift domain are identical across the three simulations, reflecting identical stochastic collisions under the identical IMS conditions. This illustrates the deterministic reproducibility of the stochastic simulation workflow when the input configuration and the RNG seed value are fixed. The trajectories diverge only in the quadrupole domain because of the different RF and DC voltages applied in the simulations. The multidomain results demonstrate that ICARION can simulate the motion of individual ions across multiple instrument domains within a single simulation run. Each domain defines its own electric field, gas environment, and boundary conditions, while ions retain continuous trajectories across the domain interfaces. Hence, ICARION enables particle-based modeling of coupled IMS-MS instruments spanning collisional and nearly collision-free transport regimes without the manual transfer or reinitialization of ion ensembles between separate simulations.

### Performance Benchmark

To evaluate its computational performance, ICARION was benchmarked against IDSimF and SIMION on an AMD Ryzen 9 7950X CPU with 64 GB RAM. The benchmark simulated the ion mobility transport of protonated pentanal through a 20 mm drift region with a uniform electric field by using the HSS collision model. The simulations were performed in helium at 300 K and 200 Pa with a reduced electric field strength of 10 Td. The physical and numerical input parameters were closely matched among the ICARION, IDSimF, and SIMION. Minimal output settings were used to reduce the I/O overhead while still obtaining information about the final state and reproducibility.

Ensembles containing 1000, 5000, 10,000, and 50,000 ions were simulated. ICARION and IDSimF were evaluated with 1, 2, 4, 8, and 16 CPU threads at a fixed time step of 0.6 ns. For each configuration, five independent runs were performed, preceded by one warm-up run to eliminate initialization effects. The median wall-clock times were used to calculate speedup compared with single-thread execution. SIMION was evaluated in single-thread mode using adaptive integration with *T.Qual* set to 3 and a maximum time step of 0.6 ns. Since the time-step control, runtime measurement, and output handling differ from those of ICARION and IDSimF, the SIMION runtime values are considered descriptive rather than strictly equivalent.

Figure 8(a) compares the runtime in single-thread mode with that from the thread configuration that provides the lowest median runtime among the tested numbers of threads. ICARION and IDSimF exhibit similar runtimes for 1000 ions, while ICARION requires a shorter runtime for larger ensembles. When considering the fastest multithreaded configuration, ICARION consistently achieved shorter runtimes than those of IDSimF and SIMION. The fastest ICARION simulations used eight threads for all of the ensemble sizes. For IDSimF, four threads were fastest up to 10,000 ions, and eight threads were fastest at 50,000 ions. Figure 8(b) shows the parallel speedup of the ICARION simulations. With eight threads, ICARION achieves speedups of approximately 2.8, 4.2, 4.5, and 5.7 for 1000, 5000, 10,000, and 50,000 ions, respectively. Increasing the number of threads to 16 did not result in any further reduction in runtime, indicating saturation in the shared-memory scaling. This benchmark does not isolate the respective contributions of memory access, scheduling, synchronization, or other parallel overheads.

**Figure 8:**
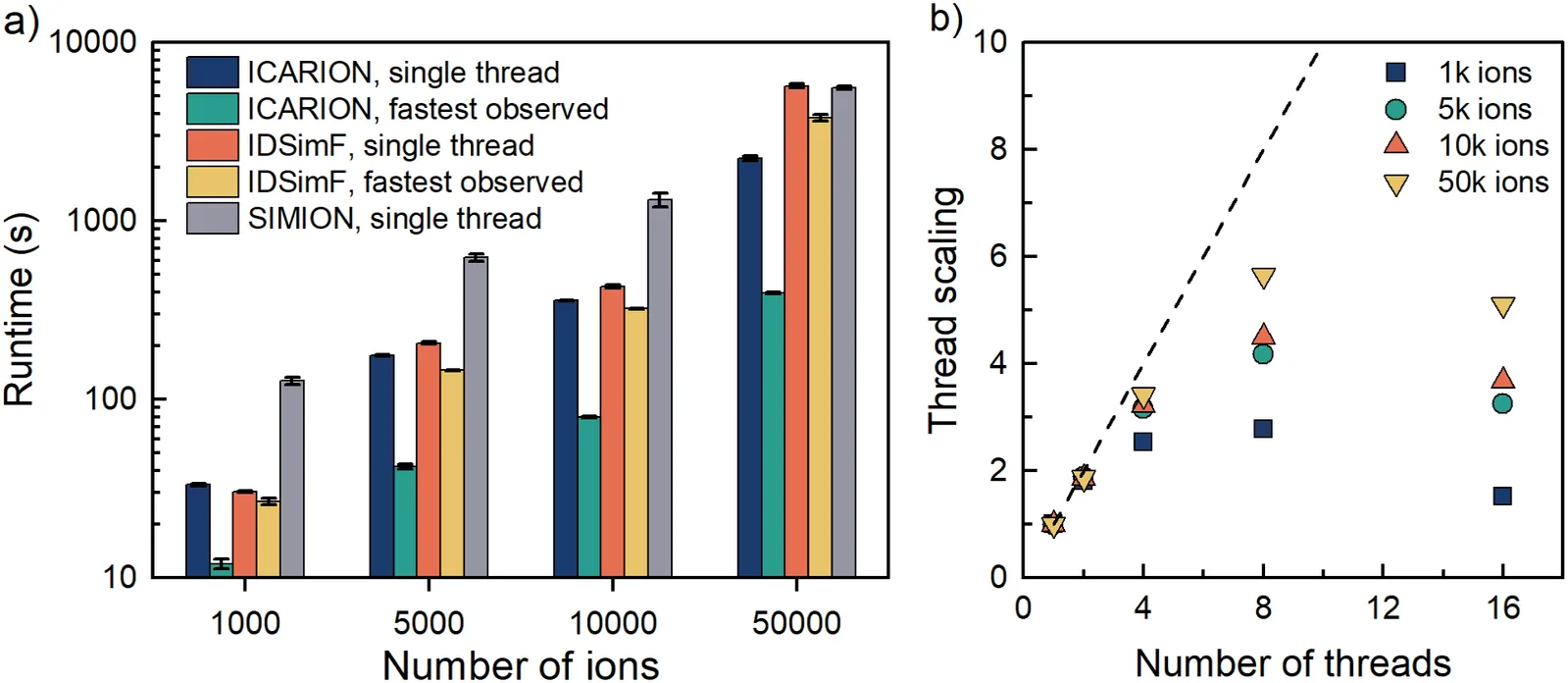
Computational performance of the single hard-sphere collision models in ion mobility simulations with ensembles ranging from 1000 to 50,000 ions. (a) Median runtime for single-thread execution and for the multithreaded configuration yielding the lowest median runtime among those tested. Error bars represent the standard deviation of five independent runs. (b) Corresponding ICARION speedup relative to single-thread execution. The dashed line indicates ideal linear scaling. Scaling improved with increasing ensemble size and reached its lowest runtime at eight threads, whereas 16 threads did not provide further acceleration. The results characterize the investigated hardware, software versions, and simulation settings and are not intended as a general performance evaluation of the three tools.

The maximum memory usage is summarized in Table 2. In the fastest configuration, ICARION’s maximum resident set size (RSS) for the ensemble sizes under investigation increased from 22.0 to 170.9 MiB, compared to 18.0 to 81.4 MiB for IDSimF. Thus, ICARION’s shorter runtimes for the larger ion ensembles were accompanied by higher memory usage.

**Table 2:** Median Maximum Memory Usage for the Thread Configuration Yielding the Lowest Median Runtime at Each Ion Ensemble Size^a^

Number of ions	ICARION: number of threads	ICARION: peak RSS (MiB)	IDSimF: number of threads	IDSimF: peak RSS (MiB)
1000	8	22.0	4	18.0
5000	8	34.3	4	23.3
10,000	8	49.0	4	29.8
50,000	8	170.9	8	81.4

a The maximum memory usage is reported as the maximum resident set size (peak RSS), representing the maximum physical RAM occupied by the process during execution. Values are medians of five independent runs.

Overall, the results show that the modular ICARION framework remains computationally feasible for the ion ensemble sizes examined and benefits from shared-memory parallelization. This benchmark refers to the specific hardware, software versions, and simulation settings used here and is not intended as a general performance evaluation of the three tools.

### Current Implementation and Future Directions

While the present validation focuses on collision models, reaction kinetics, ion transport in drift cells, and a coupled IMS-quadrupole configuration, the scope of ICARION is not restricted to ion mobility simulations or to the examples presented in this work. The same domain-based architecture can also be applied to other ion optics and mass spectrometry devices, including linear ion traps, SIFT, Orbitraps, FT-ICR, and time-of-flight regions, using available analytical field descriptions, imported field maps, and configurable time-dependent voltage waveforms. ICARION therefore provides a universal framework for particle-based ion trajectory simulations beyond ion mobility alone.

The stochastic hard-sphere collision models implemented in version v1.0.2, as used in this work, provide computationally efficient runtime descriptions for ion trajectory simulations in multidomain instruments. Starting with version v1.1.0, ICARION also offers a collision model based on explicit ion-neutral interaction potentials. However, a detailed validation of this model is beyond the scope of this work. Future work could focus on parametrizing the effective GR-HSS contact distances using reference scattering data to more closely connect the geometric model with the collision model based on interaction potentials. The currently available collision models do not account for diffuse re-emission, thermal accommodation, or transfer of translational energy to rotational, vibrational, or other internal degrees of freedom. They therefore represent an intermediate level of physical description that avoids empirical energy accommodation models while remaining computationally feasible for larger ion ensembles, in contrast to fully atomistic molecular dynamics simulations.

A major future direction of development is the integration of an internal numerical field solver into ICARION trajectory simulations. This will enable the calculation of electric fields in the same framework using user-defined electrode geometries rather than relying on analytical expressions or externally generated field maps. Complete coupled IMS-MS instruments, as well as user-defined trapping, focusing, ion-guiding, or mass-analysis stages, could then be defined within the same infrastructure for trajectories, collisions, reactions, configurations, and outputs without requiring manual transfer of ion ensembles between separate tools or simulation runs. This would also support the automated optimization of electrode geometries, voltages, pressures, time sequences, or gas compositions using objective evaluation metrics such as transmission, resolving power, confinement, energy distributions, or reaction yields.

## Conclusion

In this work, we introduce ICARION, a modular and reproducible simulation framework for particle-based ion transport across heterogeneous IMS-MS instruments. Rather than proposing new microscopic interaction models, this study demonstrates that deterministic electric fields, stochastic ion-neutral collisions, and chemical reactions can be treated consistently within a single execution framework that covers multiple pressure and electric field ranges. The implemented models reproduce the expected behavior in thermalization from collisions, bimolecular reaction kinetics, ion transport simulations validated using established simulation tools, and an example of a coupled IMS–quadrupole system.

The particle-based approach also resolves nonequilibrium transport when the collisional relaxation times become comparable to residence times in the instrument. The low-pressure drift simulations demonstrate how the obtained apparent ion mobility can maintain sensitivity to ion initialization under conditions of slow equilibration. A key strength of ICARION is its ability to simulate coupled instrument domains within a single simulation by consistently applying the same physical models under strongly varying conditions. The multidomain example with IMS and quadrupole demonstrates that ion mobility separation and RF-based mass filtering can be combined seamlessly while preserving continuous trajectories across significantly different pressure and electric field ranges without the need to manually transfer ion distributions between separate simulations or perform sequential postprocessing. This reflects the architectural requirements of modern, multistage IMS-MS instrumentation.

ICARION is primarily intended as a framework for simulating ion trajectories to propagate ion ensembles at the instrument level in electric fields and gaseous environments, rather than as a standalone tool for predicting collision cross sections or ion mobilities. Its modular architecture supports mechanistic investigations of ion dynamics, the exploration of new concepts in IMS, MS, and related techniques, and the rapid and reproducible screening of operating parameters and geometries for instrument development and optimization. Furthermore, it enables the future implementation of an internal numerical field solver without changing the general architecture or the underlying physical models.

ICARION is released as open-source software together with fully reproducible configuration and analysis workflows. By providing both prebuilt executable releases for users who wish to run simulations directly and the full source code for users who wish to inspect, modify, or extend the framework, we aim to facilitate adoption, transparency, and further development within the IMS-MS research community.

## Supplementary Material



## Data Availability

The data underlying this study are openly available in the National Data Repository at DOI: 10.48700/6t3q1-es569.^24^ These data include all simulated data sets shown in this work, together with the configuration files required for full reproducibility, all analyzed data, and all used pre- and postprocessing scripts.

## Notes

Additional details on the ICARION software architecture, implementation, physical models, numerical integration schemes, example configurations, and additional ion mobility benchmark data (PDF)
