# Supplementary material for: ICARION: A Modular Framework for Ion Trajectory Simulation in Electric Fields and Neutral Gas Environments
Source: J Am Soc Mass Spectrom. 2026 Sep 3;37(10):2483–96. doi: 10.1021/jasms.6c00235 (PMC13651595; doi:10.1021/jasms.6c00235)
Supplement: Supplementary file 1 [file js6c00235_si_001.pdf]

Supporting Information

ICARION: A Modular Framework for Ion Trajectory Simulation in Electric Fields and Neutral Gas Environments

Christoph Schaefer*, Juraj Jašík, Patrik Španěl

Heyrovský Institute of the Czech Academy of Sciences, Dolejškova 3, 18223 Prague 8, Czech Republic

Corresponding Author's email address: christoph.schafer@heu.cas.cz

Table of Contents

S1. Software Architecture 1
High-level Architecture 1
Core Components 1
Strategy & Factory Pattern 2
Module Overview 2
S2. Implementation Details 4
Domain and Field Architecture 4
CPU Parallelization 5
Simulation Configuration (JSON/SSOT) 6
HDF5 Output and Reproducibility Metadata 7
S3. Physical Models and Numerical Integration 8
Collision Handling 8
Space Charge Modeling 17
Numerical Integration 18
S4. Example Configurations - Overview 19
Thermalization Case 20
Reaction Kinetics Case 22
Ion Mobility Configuration 23
Multi-Domain Case 25

Performance Benchmark 26
S5. Additional Thermalization Data 28
S6. Additional Ion Mobility Data....................30
References 31

S1. Software Architecture

This section provides a technical reference for the ICARION software architecture and the physical models used in the present work. It is intended as supplementary documentation to support transparency and reproducibility and is not essential for understanding the validation results discussed in the main manuscript. All simulations were generated from Git commit 40210bfe, which contains the code subsequently released unchanged in ICARION v1.0.2. The final v1.0.2 release commit (0f5dfce3) also includes regenerated precomputed data and test suite corrections that do not change the relevant simulation code used in the reported simulations in the main manuscript.

High-level Architecture

ICARION's high-level architecture follows a strict separation of concerns. Numerical integrators are independent of the active physics models and domain definitions; physical model implementations are independent of the chosen integrator; and geometries, fields, and environmental parameters are defined separately from both. Internally, a structure-of-arrays (SoA) data layout is used to support efficient iteration in the performance-critical CPU loop. Factories, registries, and stable interfaces are employed to enable interchangeable model selections while maintaining clear boundaries between responsibilities.

Core Components

The main software modules and their interfaces are organized hierarchically as shown in the schematic listing below.

```
SimulationEngine
 │
 ├─ DomainManager
 │      ├─ Geometry (IDomainGeometry: Cylindrical, Orbitrap)
 │      └─ FieldModel (IFieldModel: AnalyticalFieldModel, FieldProviderModel)
 │
 ├─ Physics
 │      ├─ CollisionHandler (HSS, GR-HSS)
 │      ├─ ReactionHandler (StochasticReactionHandler, NoReactionHandler)
 │      └─ ForceRegistry (IForce: E, B, Damping, space charge via ISpaceChargeModel)
 │
 ├─ Integrators (RK4, RK45, Boris)
 │
 ├─ SpaceCharge (Direct, Grid/Poisson)
 │
 └─ I/O
        ├─ ConfigLoader (JSON → FullConfig)
        └─ OutputManager (HDF5 + reproducibility metadata)
```

At its core, ICARION consists of interacting subsystems coordinated by the `SimulationEngine`, which orchestrates the time step loop and the overall control flow. Domain handling is managed by the `DomainManager`, which allows instruments to be composed of one or more ordered domains. Physics modules include the `CollisionHandler`, `ReactionHandler`, and `ForceRegistry`, which provide stochastic collisions, chemical reactions, and deterministic forces. Numerical integration is performed using interchangeable `IntegratorStrategy` implementations. In v1.0.x, the primary validated execution path is CPU-based; GPU helper code is compiled, but GPU execution is disabled at runtime. The I/O subsystem manages configuration parsing, data and metadata logging, and structured HDF5 output with configuration snapshots.

Strategy & Factory Pattern

ICARION applies strategy-based abstractions and factory mechanisms throughout the codebase to support extensibility and to clearly separate numerical integrators from physical models. Collision handlers, reaction handlers, integration strategies, boundary actions, and space charge solvers provide stable interfaces and can be selected or instantiated through dedicated factory classes where applicable. Domain geometries and field models are constructed during domain setup and are managed by the `DomainManager`.

This design allows new physical models or numerical integrators to be added without modifying the main `SimulationEngine` or other subsystems, while keeping the same execution logic for different model implementations.

Module Overview

ICARION is organized into modular subsystems with clearly defined responsibilities and strictly separated concerns. A schematic overview of the primary modules instantiated during a simulation run is shown in Figure S1.

1. The `DomainManager` defines the geometric boundaries and physical properties of each domain by linking a geometry (`IDomainGeometry`) with an electric field model (`IFieldModel`, such as `AnalyticalFieldModel` or `FieldProviderModel`). It determines which domain an ion occupies, handles boundary conditions, and controls transitions between domains.
2. The `CollisionHandler` provides stochastic collision models (HSS/GR-HSS) as well as deterministic damping models (HSD/Langevin/Friction), and applies momentum transfer for individually sampled stochastic collisions.
3. The `ReactionHandler` samples chemical reactions using user-defined rate coefficients and updates the ion species accordingly (`StochasticReactionHandler` or `NoReactionHandler`). Temperature-

dependent rate expressions and related settings are specified in the `ReactionConfig`.

4. The `ForceRegistry` aggregates all active force contributions (electric, magnetic, and optional collisional drag) and provides the combined force vector used by the integrator. Forces resulting from space charge fields are provided through an attached `ISpaceChargeModel`.
5. The `IntegratorStrategy` implements the numerical method used to propagate ion motion (e.g., RK4, RK45, Boris pusher, see Section S3 for details). Integrators are independent of the physical model implementations and operate only on positions, velocities, and the combined force vector.
6. The optional `SpaceChargeModel` computes electric fields arising from ion-ion interactions using direct or grid-based approaches.
7. The `ConfigLoader` constructs a fully traceable single-source-of-truth (SSOT) configuration from the JSON input, while the `OutputManager` writes trajectories, metadata, logging information, and reproducibility records to an HDF5 file.
8. Finally, the `SimulationEngine` coordinates the time step loop and controls the execution order of domain resolution, collision sampling, reaction sampling, force evaluation, numerical integration, boundary handling, and conditional logging.

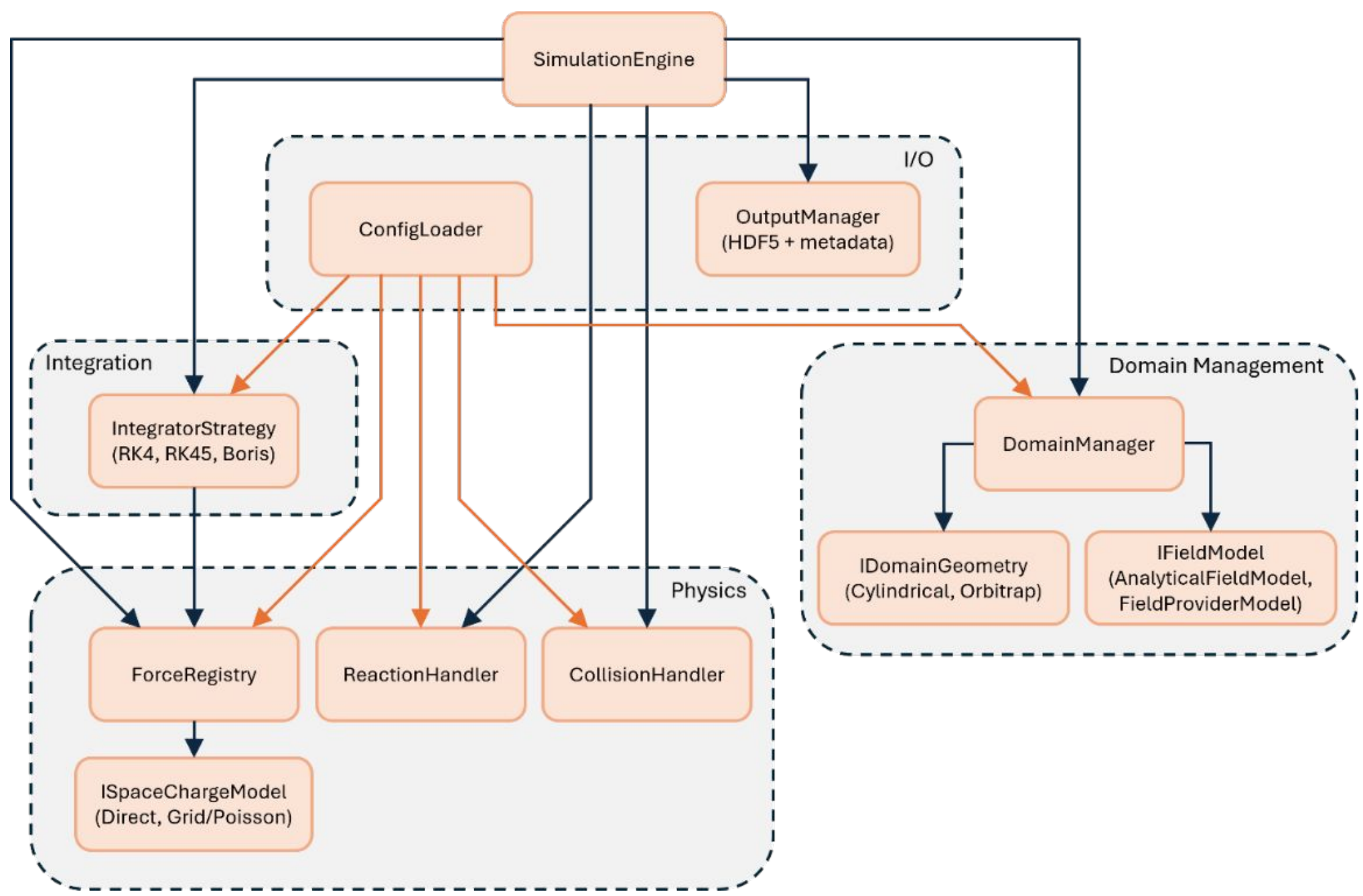

Figure S1. Schematic overview of the ICARION software architecture and module interactions. The `SimulationEngine` serves as the central orchestrator, coordinating domain handling, physical models, numerical integration, and I/O operations. Instrument geometries, electric fields, and environmental parameters are managed by the `DomainManager`. Physical processes such as collisions and reactions are implemented through dedicated handlers, while force contributions are aggregated via the `ForceRegistry`. Numerical integration is performed by interchangeable integrator strategies. Configuration input and structured HDF5 output, including reproducibility metadata, are handled by the I/O subsystem. The figure illustrates the separation of concerns and the modular design underlying ICARION.

S2. Implementation Details

Domain and Field Architecture

In ICARION, a domain represents a spatial region within the instrument with a distinct geometry, electric field definition, and environment (pressure, temperature, gas composition, and optional bulk gas flow). Domains provide the fundamental unit for separating different instrument regions and handling boundaries, allowing a single simulation run to combine heterogeneous regions such as drift, trapping, and m/z analysis stages.

The `DomainManager` maintains all active domains and determines which domain contains a given ion position. Ion positions are propagated in a global Cartesian coordinate system. Each domain is placed within this global frame through a defined local origin and orientation, so that each domain can use its own local coordinate systems while ICARION keeps a unified global representation for propagation and logging.

Each domain is defined by three components:

1. **Geometry (`IDomainGeometry`)**
   ICARION currently supports:
   - Cylindrical domains (`CylindricalGeometry`) defined by axial length and radius, and
   - Orbitrap geometries (`OrbitrapGeometry`) defined by inner radius, outer radius, and characteristic radius.

   Geometry objects are constructed during domain setup and provide the boundary checks and geometric information required during propagation.

2. **Electric field model (`IFieldModel`)**
   Electric fields can be defined either as:
   - analytical models (`AnalyticalFieldModel`), based on idealized field expressions, or
   - imported field maps (`FieldProviderModel`), loaded from external solvers via HDF5.

   Analytical models follow standard idealized theory (summarized in Table S1). The only explicit simplification used in the analytical set is the LQIT axial potential, which is approximated by a harmonic function; other analytical models retain their idealized expressions. Field components are defined in the domain's local frame (axial along local z, radial in local x/y) and are transformed consistently into the global frame during evaluation. Imported numerical fields are interpolated from HDF5 datasets at runtime via the `FieldProviderModel.`

3. **Environment (`EnvironmentConfig`)**
   Each domain defines its own environment parameters, including pressure, temperature, gas composition, and optional bulk gas flow. This supports explicit discontinuities across boundaries (e.g., pressure interfaces).

Table S1. Summary of analytical electric field models supported in ICARION.

Domain specification	Description
Ion mobility spectrometer (IMS)	1. Constant axial electric field in the drift tube<br>2. (Optional) quadrupolar field for radial ion confinement
Time-of-flight (TOF)	1. Constant axial electric field in the acceleration region
Quadrupole mass filter	1. Radial quadrupolar field from RF and DC voltages between adjacent quadrupole rods<br>2. Constant axial field across the entire length
Linear quadrupole ion trap (LQIT)	1. Radial quadrupolar field from RF and DC voltages between adjacent electrodes<br>2. (Simplified) harmonic axial potential from DC endcap voltages<br>3. (Optional) AC dipolar excitation in the x dimension
Orbitrap	1. Radial electric field following a quadro-logarithmic potential between inner and outer electrodes<br>2. Axial electric field linear in z (harmonic potential), derived from the quadro-logarithmic Orbitrap potential
Fourier transform ion cyclotron resonance (FTICR)	1. Electrostatic quadrupolar trapping potential<br>2. Static magnetic field (user-defined, usually aligned with z)

A representative multi-domain setup used in the main manuscript is the IMS-quadrupole configuration (see the associated dataset in the National Data Repository[1] for the configuration file). In this setup, two domains (the drift cell and the quadrupole) are connected sequentially along the global z-axis via a virtual aperture. Ions are generated in the IMS domain and transition into the quadrupole upon crossing the shared interface. Domain-specific fields and environments govern the subsequent ion motion. If an ion exits a domain outside the defined apertures, it is either removed from the simulation through absorbing boundaries or reflected, depending on the specified boundary condition.

CPU Parallelization

ICARION uses OpenMP to parallelize per-ion operations on multi-core CPUs, including numerical integration, stochastic collision sampling, and reaction sampling. A SoA

memory layout stores frequently accessed ion properties (position, velocity, mass, charge) in contiguous memory blocks, improving cache locality and enabling efficient vectorization.

To avoid contention during parallel execution, each ion maintains an independent random number generator (RNG) stream using a deterministic seed scheme (global seed + ion index). This avoids shared RNG across threads and ensures deterministic behavior under parallel execution.

The CPU is the primary validated execution path in v1.0.x. Achievable scaling depends on workload composition, and shared-memory scaling may saturate at higher thread counts. The present benchmark does not isolate the limiting component.

Simulation Configuration (JSON/SSOT)

All simulations are specified through a JSON-based configuration system following a strict SSOT principle. Simulation parameters are taken directly from the input files, and derived quantities (e.g., neutral number density from pressure and temperature, or field parameters) are computed deterministically from these inputs. The resolved configuration, therefore, provides a complete and traceable description of each run.

Up to five JSON input files may be used in a simulation run:

1. **Simulation configuration file**
   Defines global simulation parameters (total simulation time, time step resolution), domain definitions (instrument type, geometry parameters, field definitions, environmental conditions), solver settings, and output options (paths, write intervals). Multi-domain instruments are defined as ordered domain sequences that enable seamless transitions between drift, trapping, and time-of-flight regions within a single run.
2. **Species database**
   Contains physical and chemical properties (charge state, ion mass, reduced ion mobility and/or collision cross section, optional polarizability) and may reference molecular structure files.
3. **Reaction database**
   Specifies reaction channels, associated rate coefficients, and optional Arrhenius or modified Arrhenius (Kooij) parameters.

4. **Ion cloud file (optional)**
   Provides explicit initial ion ensembles when not fully defined in the simulation configuration. If defined in the configuration, supported spatial distributions include point sources and uniform or Gaussian clouds. Velocity distributions may be fixed, sampled from Maxwell distributions, drawn from Gaussian distributions, or derived

from user-defined kinetic energies. Multi-species injections are supported by assigning separate distributions and ion counts per species.

5. **Molecular structure files (optional)**
   Provide atomic-level molecular geometries (typically from DFT optimization) required for structure-resolved collision models and referenced via the species database.

Not all files are required for every run. A species database is required for any simulation that includes ions, while a reaction database is required only if reactions are enabled. Ion clouds and molecular geometries are optional. If run-specific species or reactions are not required, shared central species and reaction databases serve as fallbacks.

During parsing, ICARION loads core configuration sections, computes derived quantities, and then resolves external database paths. All configuration files are validated prior to execution, with critical errors raising exceptions and non-blocking issues reported as warnings. Ion cloud files are validated during ion loading.

HDF5 Output and Reproducibility Metadata

Each simulation run generates a dedicated HDF5 output file with its path specified in the configuration. The HDF5 hierarchy stores simulation results and associated metadata in a self-contained format, including per-ion trajectory data (position, velocity, time), global simulation parameters, domain definitions, and complete metadata for all species and reactions used in the simulation. To balance I/O overhead and temporal resolution, output is written using a buffered output system with configurable write intervals and an optional maximum buffer size. Trajectory records are accumulated in memory and flushed to disk at scheduled write times or when buffer limits are reached, reducing file system overhead while keeping consistent output timing.

For reproducibility, ICARION stores runtime metadata in the HDF5 file, including the RNG seed scheme (global seed + ion index), compiler and build information, operating system details, and the Git commit hash identifying the exact code version. Input file hashes are recorded, and the HDF5 metadata can embed copies of relevant inputs (configuration/species/reaction JSON and, where applicable, field arrays) for traceability. In addition, a snapshot of the resolved JSON configuration is written to the output directory, enabling reproducible reruns across systems and over time.

S3. Physical Models and Numerical Integration

Collision Handling

ICARION supports both deterministic and stochastic descriptions of ion-neutral collisions. Deterministic models approximate the cumulative effect of many individual collisions through a continuous drag force added to the equations of motion, whereas stochastic models explicitly sample discrete collision events via Monte Carlo methods. Only one collision model is active in each simulation; deterministic and stochastic approaches are therefore mutually exclusive.

Deterministic Collision Models

The deterministic damping force is described by eq. (S1), where m_{ion} is the ion mass, γ a model-dependent damping coefficient, and $\vec{v}$ is the ion velocity $\vec{v}_{\mathrm{ion}}$ relative to the bulk gas flow velocity $\vec{v}_{\mathrm{gas}}$. This formulation reproduces average drift behavior and steady-state mobilities but does not capture diffusion, thermalization, or stochastic velocity fluctuations.

$$\vec{F}_{\mathrm{coll}} = -\,m_{\mathrm{ion}}\gamma\vec{v} = -\,m_{\mathrm{ion}}\gamma(\vec{v}_{\mathrm{ion}} - \vec{v}_{\mathrm{gas}}) \quad \text{(S1)}$$

Hard-Sphere Deterministic Model

In the hard-sphere model, collisions are assumed to occur with a constant geometric cross section σ_{geom}. The damping coefficient follows directly from kinetic theory (eq. (S2)), where N is the neutral number density and v_{th} the mean thermal velocity of the background gas. This model provides a simple estimate of momentum transfer when short-range repulsive interactions dominate.

$$\gamma = N\sigma_{\mathrm{geom}}v_{\mathrm{th}} \quad \text{(S2)}$$

Langevin Model

For polarizable neutral gases, ICARION provides an approximate velocity-dependent deterministic damping model based on the Langevin capture cross section. In the implementation of ICARION v1.0.x, the cross section depends inversely on the instantaneous ion speed (eq.). (S3)), where q is the ion charge, α the neutral polarizability, ϵ_0 the vacuum permittivity, and μ the reduced mass of ion and neutral. The damping coefficient additionally contains the mean thermal speed of the background gas. Individual neutral velocities are not sampled in this deterministic model.

$$\sigma_{\mathrm{L}} = \pi q \sqrt{\frac{\alpha}{4\pi\epsilon_0 \mu}} \frac{1}{|\vec{v}|} \quad \text{(S3)}$$

Friction (Mobility-Enforcing) Model

The friction model enforces a prescribed ion mobility by choosing γ such that the steady-state drift velocity satisfies $\vec{v}_D = K\vec{E}$. By equating this with the force balance implied by eq. (S1), γ can be described by eq. (S4). The ion mobility itself is computed from the reduced ion mobility K_0 (eq. (S5)) with the Loschmidt number N_0.

$$\gamma = \frac{q}{m_{\mathrm{ion}} K} \quad \text{(S4)}$$

$$K = K_0 \frac{N_0}{N} \quad \text{(S5)}$$

Applicability and Limitations

Deterministic collision models are computationally efficient and suitable for simulations that require only average transport properties. However, by replacing discrete collisions with a continuous force, they inherently neglect stochastic momentum transfer. As a result, diffusion, thermalization, and velocity fluctuations cannot be reproduced. These effects are essential for detailed IMS simulations, particularly at low pressures and in systems where diffusive broadening and non-equilibrium effects are relevant. In such cases, stochastic collision models should be employed.

Monte-Carlo Collision Sampling

When a stochastic collision model is enabled, the motion of neutral collision partners is not explicitly calculated at the instrument level. Instead, for each collision calculation, the velocity of a potential neutral collision partner is drawn from the local Maxwellian distribution, including the bulk gas velocity. Each Cartesian component $v_{\mathrm{n},j}$ is drawn independently from a normal distribution according to eq. (S6), where $u_{\mathrm{gas},j}$ is the corresponding component of the bulk gas velocity, m_{n} is the neutral mass, and ξ_j are independent, normally distributed random numbers. This component-wise sampling yields the corresponding Maxwellian speed distributions for the neutral ensemble.

$$v_{\mathrm{n},j} = u_{\mathrm{gas},j} + \sqrt{\frac{k_{\mathrm{B}} T}{m_n}} \xi_j \quad \text{(S6)}$$

The relative velocity is then calculated using eq. (S7).

$$\vec{v}_{\mathrm{rel}} = \vec{v}_{\mathrm{ion}} - \vec{v}_{\mathrm{n}} \quad \text{(S7)}$$

In the following, σ_{event} denotes the collision event cross section used to determine the stochastic collision rate. The instantaneous collision rate k_{coll} is calculated according to eq. (S8), where N is the neutral number density and $|\vec{v}_{\mathrm{rel}}|$ the instantaneous relative speed between ion and neutral. Assuming a Poisson process, the probability that at least one collision occurs within a time step Δt is given by eq. (S9). A uniformly distributed random number $u \in [0,1]$ is drawn, and the collision is accepted if $u < P_{\mathrm{coll}}$. If a collision is accepted, the same drawn neutral velocity that was used to calculate the collision rate is used for the subsequent calculation of the collision and the associated update of the ion velocity. The neutral velocities are drawn independently at each collision evaluation.

$$k_{\mathrm{coll}} = N\sigma_{\mathrm{event}}|\vec{v}_{\mathrm{rel}}| \tag{S8}$$

$$P_{\mathrm{coll}} = 1 - \exp\left(-k_{\mathrm{coll}}\Delta t\right) \tag{S9}$$

Multi-Gas Collision Kinetics

For gas mixtures, a neutral velocity is drawn independently for each active gas component i. The corresponding partial collision rate is given by eq. (S10), where N_i is the component-specific number density and $\sigma_{\mathrm{event},i}$ is the collision event cross section. The partial rates are summed to obtain the total collision rate according to eq. (S11). If a collision is accepted, the gas component is selected with a probability given by eq. (S12). The previously drawn neutral velocity of the selected gas component is then used to update the ion velocity.

$$k_i = N_i\sigma_{\mathrm{event},i}|\vec{v}_{\mathrm{rel},i}| \tag{S10}$$

$$k_{\mathrm{tot}} = \sum_i k_i \tag{S11}$$

$$P_i = \frac{k_i}{k_{tot}} \tag{S12}$$

Effective Single-Sphere Hard-Sphere Scattering (HSS) model

In the HSS model, the ion-neutral collisions are described using an effective single-sphere model. The model requires neither an explicit ion geometry nor an ion-neutral interaction potential and therefore provides a computationally efficient approximation for rapid screening and simulations of large ion ensembles. The specified σ_{event} value determines the stochastic collision rate according to eq. (S8).

For the validation cases presented in this work, the CCS calculated with MobCal-MPI 2.0[2] for low-field conditions was used as σ_{event}. In the trajectory method implemented in MobCal-MPI, scattering trajectories are calculated as functions of the relative speed between the ion and the neutral, the impact parameter, and the relative orientation between them, using an explicit ion-neutral interaction potential. This potential includes

short-range repulsive and dispersion interactions, as well as the long-range, attractive ion-induced dipole interaction. The resulting scattering angles are integrated over the impact parameter and averaged over the relative orientations between ion and neutral to obtain momentum transfer cross sections as a function of collision energy. Subsequently, a transport-weighted averaging is performed over the distribution of relative velocities between ion and neutral to obtain the first-order collision integral $\Omega^{(1,1)}$. [2]

Using this thermally averaged transport quantity as σ_{event} condenses the underlying dependence on collision energy, impact parameter, relative orientation between the ion and the neutral, and the interaction potential into an effective single-sphere description. This is an intentional approximation that reduces the computational cost. It can reproduce ensemble-averaged transport behavior when the provided CCS is representative of the conditions under investigation, but it does not preserve the underlying dependence of the ion-neutral collision on collision energy.

Once a collision is accepted, the event is treated as a binary elastic collision between two effective hard spheres in the center-of-mass reference frame. The center-of-mass velocity $\vec{v}_{\mathrm{COM}}$ is calculated from the velocities of the ion and neutral, $\vec{v}_{\mathrm{ion}}$ and $\vec{v}_{\mathrm{n}}$, and their masses, m_{ion} and m_{n}, according to eq. (S13) .

$$\vec{v}_{\mathrm{COM}} = \frac{m_{\mathrm{ion}}\vec{v}_{\mathrm{ion}} + m_{\mathrm{n}}\vec{v}_{\mathrm{n}}}{m_{\mathrm{ion}} + m_{\mathrm{n}}} \tag{S13}$$

The magnitude of the relative velocity between ion and neutral is conserved during the elastic collision, while its direction after the collision is randomly sampled isotropically. Two independent random numbers u_1,u_2, which are uniformly distributed in [0,1), are used to generate the polar angle χ and the azimuthal angle ϕ according to equations (S14) and (S15). The determined angles define the unit vector $\vec{e}$ of the outgoing relative velocity, which is given in spherical coordinates by eq. (S16). The relative velocity vector $\vec{v}'_{\mathrm{rel}}$ after the collision is then determined by multiplying this unit direction by the magnitude of the incoming relative velocity according to eq. (S17). The uniformly distributed sampling of $\cos(\chi)$ instead of χ itself ensures that all directions on the unit sphere are equally likely.

$$\cos(\chi) = 2u_1 - 1 \tag{S14}$$

$$\phi = 2\pi u_2 \tag{S15}$$

$$\vec{e} = \begin{pmatrix} \sqrt{1-\cos^2(\chi)}\cos(\phi) \\ \sqrt{1-\cos^2(\chi)}\sin(\phi) \\ \cos(\chi) \end{pmatrix} \tag{S16}$$

$$\vec{v}'_{\mathrm{rel}} = |\vec{v}_{\mathrm{rel}}|\vec{e} \tag{S17}$$

Finally, the ion velocity after the collision is transformed back to the laboratory reference frame $\vec{v}'_{\mathrm{ion}}$ according to eq. (S18).

$$\vec{v}'_{\mathrm{ion}} = \vec{v}_{\mathrm{COM}} + \frac{m_{\mathrm{n}}}{m_{\mathrm{ion}} + m_{\mathrm{n}}} \vec{v}'_{\mathrm{rel}} \tag{S18}$$

In the case of elastic scattering between two smooth, hard spheres, this approach statistically corresponds to uniformly sampling the point of impact across the entire collision disk and calculating the corresponding specular reflection. Thus, the implementation directly samples the scattering distribution of the hard spheres rather than explicitly determining a point of impact for each collision. The implementation conserves the total linear momentum and the total translational kinetic energy. Isotropic scattering in the center-of-mass reference frame has also been used in other Monte Carlo methods for hard-sphere simulations of ion mobility and diffusion.[3]

Geometry-Resolved Single-Contact Hard-Sphere Scattering (GR-HSS) Model

In the GR-HSS model, the ion is represented by a rigid three-dimensional geometry composed of atom-centered hard spheres. The contact geometry between the atom and helium is defined using the sum of van der Waals radii from the internally consistent dataset of Mantina et al.[4] For atom i of the ion and a helium atom, the center-to-center distance $d_{i,\mathrm{He}}$ is therefore given by eq. (S19), where r_i and r_{He} are the corresponding van der Waals radii.

$$d_{i,\mathrm{He}} = r_i + r_{\mathrm{He}} \tag{S19}$$

To reduce runtime computational load, the ion orientations and their corresponding projected collision areas are calculated in advance. Similar geometric hard-sphere approaches have already estimated collision cross sections by averaging the projected areas over randomly selected orientations.[5-7] The ion orientations are sampled uniformly in three-dimensional rotational space and stored as quaternions. For each orientation, the ion geometry is projected onto a plane perpendicular to the collision axis. The projected σ_{event} is estimated by Monte Carlo sampling of the points of impact on a disk that encloses the entire projected hard-sphere geometry, whereby the fraction of detected rays that intersect at least one contact sphere of the atom and helium is determined. The resulting orientation-dependent projected areas are stored along with their corresponding orientations and used during trajectory simulation at runtime.

At runtime, one of the precomputed ion orientations is selected at random. The corresponding projected area is used as the collision event cross section σ_{event} in eq. (S8). Orientations with larger projected areas therefore have higher collision probabilities and occur more frequently among the accepted collision events. If a collision event is accepted, the same orientation is used for the scattering calculation.

The actual contact between the ion and neutral is then determined at runtime. The selected ion orientation is transformed into a coordinate system whose collision axis is aligned with the instantaneous relative velocity between ion and neutral. The impact points are sampled uniformly over a disk that encloses the projected collision geometry, using $b = b_{\max}\sqrt{u_1}$ and $\phi = 2\pi u_2$, where u_1 and u_2 are uniformly distributed random numbers. Samples that do not intersect the geometry of the hard spheres are discarded. For an accepted impact trajectory, intersection points with all atom-centered hard spheres are considered, and the earliest intersection point along the incoming trajectory is selected as the first contact between the ion and neutral.

The incoming relative velocity is reflected once at the local contact normal $\vec{n}$ according to eq. (S20).

$$\vec{v}'_{\mathrm{rel}} = \vec{v}_{\mathrm{rel}} - 2(\vec{v}_{\mathrm{rel}} \cdot \vec{n})\vec{n} \tag{S20}$$

The resulting ion velocity is then transformed back into the laboratory reference frame using the same center-of-mass transformation as in the HSS model (eq. (S18)). The ion orientation, the impact geometry, the detection of the first contact between ion and neutral, and the reflected relative velocity are all treated in three dimensions. The two-dimensional illustration in Figure 2 of the main manuscript is therefore merely schematic.

GR-HSS considers only the first geometric contact, followed by a single elastic specular reflection. Secondary contacts and multiple scattering in concave ion geometries are not accounted for. It therefore differs from the conventional exact hard-sphere scattering model,[8] which explicitly includes multiple scattering.

Model Comparison and Applicability

The HSS and GR-HSS models use the same framework stochastic event selection but employ σ_{event} values with different physical origins: a CCS value derived from MobCal-MPI in HSS, and an orientation-dependent projected area in GR-HSS. These quantities should therefore not be interpreted as alternative representations of the same CCS. While the instantaneous relative speed between ion and neutral enters the collision rate, the σ_{event} values used by HSS and GR-HSS are independent of collision energy.

Both hard-sphere models describe elastic collisions. HSS does not contain explicit polyatomic geometry, whereas GR-HSS represents the ion as a rigid polyatomic structure. The collision model introduced in ICARION v1.1.0, which is based on explicit ion-neutral interaction potentials, also uses fixed molecular geometries in individual scattering calculations. However, through the underlying interaction potential, it also accounts for the collision energy dependence of ion-neutral scattering.

HSS and GR-HSS do not account for diffuse re-emission, thermal accommodation, or the transfer of translational energy to internal degrees of freedom. The diffuse hard-sphere

scattering (DHSS) model[9, 10] provides an alternative physical description that can incorporate non-specular surface re-emission and energy accommodation. DHSS therefore differs from the elastic HSS and GR-HSS models in terms of its assumptions regarding angular scattering and energy transfer. Accordingly, the increase in drag force described by Epstein[11] – which is associated with diffuse and thermally accommodating re-emission – does not represent an expected limiting value for the elastic collision models used here.

The treatment of inelastic collisions would require additional models or data describing the corresponding angular scattering and energy transfer and is not included in the present ICARION collision models.

Parametrization of the GR-HSS Model

Long-range attractive ion–induced dipole interactions can lead to significant scattering at distances beyond the range of short-range repulsion. Their magnitude depends on the polarizability of the neutral, and their contribution generally decreases with increasing relative velocity between ion and neutral, as the interaction time shortens and short-range repulsion increasingly dominates the scattering. One might therefore expect potential-based and hard-sphere descriptions to converge in regimes dominated by short-range repulsion. However, the first-order collision integral obtained from MobCal-MPI and the mean GR-HSS values of σ_{event} are not identical physical quantities and are derived from different scattering laws. The collision integrals derived from MobCal-MPI, which are smaller than the mean GR-HSS values of σ_{event} observed in this work, may therefore initially appear counterintuitive.

One likely reason for this difference is the independent parametrization of GR-HSS. In ICARION v1.0.2, the element-specific contact distances between atoms and helium – which are used to determine the first contact between ions and neutrals – are determined by summing the respective van der Waals radii reported by Mantina et al.[4] This provides a single, internally consistent, literature-based set of parameters without ion-specific fitting to calculated or experimental ion mobility or CCS data. The resulting geometric parametrization is both an advantage and a current limitation of GR-HSS: While it avoids data-specific empirical fitting, the resulting hard-sphere size does not correspond to the finite repulsive interaction considered in MobCal-MPI and related trajectory methods, even when the contributions from attractive long-range forces become negligible, since the hard-sphere approach effectively assumes infinitely steep potential barriers. Consequently, differences between GR-HSS and the transport quantities derived from MobCal-MPI can be attributed both to the different scattering laws and to the independent parametrization of the hard-sphere contact geometry.

Future parameterization could determine the effective GR-HSS contact distances between atoms and helium by fitting reference data on transport or scattering, such as

first-order collision integrals or momentum transfer cross sections. It remains to be clarified whether such parameters are transferable to different ions, neutral gases, interaction potentials, temperatures, and ranges of relative velocities.

Reaction Handling

ICARION treats ion-molecule reactions using an explicit stochastic Monte Carlo framework analogous to the collision sampling scheme. Chemical reactions are evaluated independently for each ion and time step, ensuring consistent coupling between transport, collisions, and chemistry within the same time-discretized simulation loop.

Stochastic Reaction Sampling

For each ion and time step Δt, all chemically applicable reaction channels i are identified based on the current ion species and the active reaction database. For each channel i, an effective stochastic rate $k_{\mathrm{eff},i}$ is evaluated. The total effective reaction rate is then given by eq. (S21). Channels with vanishingly low total rates ($k_{\mathrm{tot}} < 10^{-60}\ \mathrm{s}^{-1}$) are skipped for numerical safety.

$$k_{\mathrm{tot}} = \sum_i k_{\mathrm{eff},i} \tag{S21}$$

The probability that any reaction occurs within the current time step is evaluated from the Poisson distribution (eq. (S22)). For large values of $k_{\mathrm{tot}}\Delta t$, the probability is numerically truncated to $P_{\mathrm{react}} \approx 1$ to avoid numerical underflow. A uniformly distributed random number $u \in [0,1]$ is drawn, and a reaction event is accepted if $u < P_{\mathrm{rxn}}$.

$$P_{\mathrm{rxn}} = 1 - \exp\left(-k_{\mathrm{tot}}\Delta t\right) \tag{S22}$$

As validated in the reaction kinetics benchmark in the main manuscript, this formulation ensures correct stochastic sampling of reaction events at small time steps and leads to the expected exponential kinetics under pseudo-first-order conditions.

Reaction Channel Selection and State Update

If a reaction event is accepted, the specific reaction channel is selected by weighted Monte Carlo sampling, with the probability given by eq. (S23). The ion species is then updated through the centralized or run-specific `SpeciesDatabase`, which serves as an SSOT for all species properties during the run. This update propagates the new ion identity, mass, charge, collision cross section, reduced ion mobility, and associated metadata consistently throughout the simulation.

$$P_{\mathrm{i}} = \frac{k_{\mathrm{eff},i}}{k_{\mathrm{tot}}} \tag{S23}$$

Thermal Rate Coefficients

For each reaction channel, the thermal rate coefficient $k(T)$ is evaluated from the selected analytical rate law prior to conversion into the effective stochastic rate k_{eff}. ICARION currently supports three classes of rate models:

(i) Constant Rate Model

For reactions without explicit temperature dependence, a constant thermal rate coefficient is used (eq. (S24)). This model is appropriate for barrierless reactions or when only temperature-averaged literature values or estimations are available.

$$k(T) = k_0 \tag{S24}$$

(ii) Arrhenius Model

For thermally activated reactions, the Arrhenius expression is employed (eq. (S25)). In this equation, A is the pre-exponential factor, E_{a} is the activation energy, k_{B} is the Boltzmann constant, and T is the ambient temperature. Activation energies are stored internally in units of eV and converted to SI units during evaluation. For numerical stability, the exponential term is explicitly clamped for extreme arguments. If $E_{\mathrm{a}} \leq 0$, the model automatically reduces to the constant-rate form.

$$k(T) = A \exp\left(-\frac{E_{\mathrm{a}}}{k_{\mathrm{B}} T}\right) \tag{S25}$$

(iii) Modified Arrhenius (Kooij) Model

To capture non-classical temperature scaling, ICARION additionally supports the modified Arrhenius expression (eq. (S26)), where n is an empirical temperature exponent and T_0 is a user-defined reference temperature. The exponential term is evaluated with the same numerical protection as in the classical Arrhenius model.

$$k(T) = A\left(\frac{T}{T_0}\right)^n \exp\left(-\frac{E_{\mathrm{a}}}{k_{\mathrm{B}} T}\right) \tag{S26}$$

Conversion to Effective Stochastic Rate

Thermal rate coefficients $k(T)$ are converted into effective stochastic reaction rates k_{eff} by applying the reaction order and the corresponding reactant concentrations. Each reaction term explicitly specifies its concentration source. If the configuration field `concentration_m3` is set to -1, the concentration is taken from the active gas mixture when available. Otherwise, the contribution is set to zero.

Runtime Safety

If an unknown rate model is encountered at runtime, ICARION terminates with a descriptive internal error to ensure strictly defined, reproducible reaction kinetics.

Space Charge Modeling

Space charge effects arise when collective Coulomb interactions between ions significantly perturb the effect of externally applied electric fields. In ICARION, space charge is treated as an optional physical contribution that can be enabled or disabled through the simulation configuration. All space charge solvers expose their electric field contributions through the unified `ForceRegistry` interface and are therefore compatible with all numerical integrators and collision models.

Unless explicitly stated otherwise, space charge is disabled in the validation cases shown in the main manuscript, as the investigated ion densities correspond to dilute ensembles for which collective effects are expected to be small. For validation of our space charge implementation, refer to the validation report for ICARION v1.0.x in our GitHub repository, archived on Zenodo.[12]

Depending on ion count and configuration, ICARION selects between two space charge solvers that balance numerical accuracy and computational cost: a direct pairwise Coulomb solver for small ensembles and a grid-based Poisson solver for larger ion ensembles.

Direct Coulomb Solver (N-body)

For simulations involving small ion ensembles, ICARION employs direct pairwise Coulomb summation to compute the space charge electric field. For each ion i, the electric field contribution from all other ions $j \neq i$ is evaluated from eq. (S27), where k_{C} is Coulomb's constant and ϵ is a softening parameter introduced to prevent numerical divergences at very small inter-ion distances. The softening term is particularly important in simulations where ions may remain in close proximity for extended periods. Without softening, such configurations could lead to unrealistically large accelerations within a single integration step.

$$E_i = \sum_{j \neq i} k_{\mathrm{C}} q_j \frac{\vec{r}_i - \vec{r}_j}{\left(|\vec{r}_i - \vec{r}_j|^2 + \epsilon^2\right)^{3/2}} \tag{S27}$$

This solver operates directly on the ion ensemble without grid discretization or interpolation, thereby avoiding grid-related discretization and interpolation errors. Since the computational cost scales as $O(N^2)$, the direct solver is automatically selected only for small ion counts (v1.0.x default: fewer than 1000 ions). In this regime, it is well-suited

for precision studies, reference calculations, and validation of other space charge implementations.

Grid-based Poisson Solver

For larger ion ensembles, where direct $O(N^2)$ summation is no longer computationally feasible, ICARION employs a grid-based Poisson solver. Ion charges are deposited onto an axis-aligned Cartesian grid constructed from the domain's bounding box, with optional padding. The grid does not conform to electrode geometries; instead, geometry-specific Dirichlet masks are applied when provided by the domain definition.

Ion charges are deposited onto the grid to obtain a discretized charge density $\rho(\vec{r})$. For standard ensemble sizes, cloud-in-cell deposition is used, distributing each ion charge to the eight surrounding grid nodes *via* trilinear interpolation. The electrostatic potential Φ is then obtained by solving Poisson's equation (eq. (S28)) using an iterative finite-difference solver. The solver implementation is selected based on the grid size, while convergence tolerance and iteration limits are adjusted based on the ion count.

$$\nabla^2\Phi = -\frac{\rho}{\epsilon_0} \tag{S28}$$

The electric field is calculated from the potential by centered finite differences and interpolated back to the ion positions using trilinear interpolation. The resulting particle-mesh formulation avoids explicit pairwise Coulomb summation and has a per-update cost of approximately $O\left(N + N_{\mathrm{iter}}N_{\mathrm{grid}}\right)$, making it suitable for moderately dense ion clouds in extended instrument geometries while retaining the long-range component of the space charge interaction.

Numerical Integration

The motion of ions in ICARION is described by Newton's equation of motion (eq. (S29) and (S30)), where $\vec{r}$ is the position, $\vec{v}$ the velocity, m_{ion} the ion mass, and $\vec{F}$ the total force assembled by the `ForceRegistry`. Depending on the configuration, this force may include contributions from external electric and magnetic fields, space charge interactions, and deterministic collisional drag.

$$\frac{\mathrm{d}\vec{r}}{\mathrm{d}t} = \vec{v} \tag{S29}$$

$$\frac{\mathrm{d}\vec{v}}{\mathrm{d}t} = \frac{\vec{F}\left(\vec{r},\vec{v},t\right)}{m_{\mathrm{ion}}} \tag{S30}$$

ICARION provides three numerical integration schemes that can be selected at runtime: a fixed-step fourth-order Runge-Kutta method (RK4), an adaptive Dormand-Prince type RK45 integrator with embedded error estimation, and a Boris pusher for motion dominated by the Lorentz force.

Fixed-Step Runge-Kutta (RK4)

The classical fourth-order Runge-Kutta scheme is the default integrator in ICARION. It offers a robust balance between numerical accuracy and computational efficiency for smooth force fields and is well-suited for electrostatic ion optics and transport simulations under collisional conditions, where the time step is constrained by the mean collision time. All force contributions are evaluated consistently through the ForceRegistry at the intermediate RK stages. The integrator operates directly on the SoA memory layout, enabling cache-efficient execution. RK4 is used in all validation cases presented in the main manuscript.

Adaptive Runge-Kutta (RK45)

For simulations requiring adaptive time step control, ICARION provides an explicit Dormand-Prince type RK45 integrator with embedded error estimation. The adaptive time step size is controlled by user-defined absolute and relative tolerances applied to both position and velocity components. This integrator is particularly useful for simulations involving strongly varying force magnitudes, transient fields, or mixed regimes where a fixed time step would be inefficient. The implementation follows standard formulations described in the literature and exploits the First-Same-As-Last property to reduce redundant force evaluations.

Boris Pusher for Lorentz Force Integration

For simulations in which magnetic fields dominate the dynamics, ICARION includes a Boris pusher integrator. This algorithm separates the electric and magnetic force contributions and performs the magnetic rotation step analytically, thereby conserving kinetic energy in the absence of electric fields and providing good long-term numerical stability. The Boris pusher is intended for simulations involving strong magnetic confinement or cyclotron motion and is not used in the validation cases discussed in the present work.

Integrator Selection and Applicability

Integrator selection in ICARION is fully controlled through the simulation configuration. In practice, RK4 is recommended for most ion mobility, collision, and reaction simulations; RK45 is advantageous when adaptive control of time step size is required; and the Boris pusher is preferred for dynamics dominated by the Lorentz force.

S4. Example Configurations - Overview

This section provides example configurations for all validation cases described in the main manuscript, including thermalization, ion-molecule reaction kinetics, ion mobility benchmarking, multi-domain simulations, and performance benchmarking. The

configuration files are fully documented and included in the associated dataset in the National Data Repository.[1] Details on the implementation and a documentation of the file structure can be found in the ICARION GitHub repository, which is archived on Zenodo.[12] All configurations comply with the ICARION v1.0.x release line. Please note that the configuration files described here use the legacy identifier "ehss" for the GR-HSS collision model.

Thermalization Case

Configuration

The general structure of an ICARION configuration file is illustrated using the thermalization validation case. The file *ehss_PentanalHp_300K_200.0Pa_comment.json* in the associated dataset provides an example configuration in which 10,000 protonated pentanal ions are propagated under field-free, collision-dominated conditions at 300 K and helium at 200 Pa without bulk gas flow.

Key settings:

- **Simulation parameters**: The simulation time is ~ 3 µs, with a time step of ~ 6 ns, corresponding to 1/50 of the mean collision time at the given pressure and temperature, using a fourth-order Runge-Kutta integrator (RK4).
- **Physical model**: Stochastic collisions using the GR-HSS model are enabled (`collision_model = "ehss"`), while reactions are disabled.
- **Ion initialization**: Ions are sampled from a thermal velocity distribution at 0.1 K and spatially centered at (0, 0, 0) with a Gaussian standard deviation of 1 mm.
- **Domain**: The simulation is conducted in a cylindrical drift cell with no electric field or gas flow. The domain spans 100 m in length and 100 m in radius to prevent ions from reaching the domain boundaries. The axial origin is shifted to – 50 m, so the domain ranges from – 50 m to + 50 m along the z-axis.

Other configurations for this validation vary in pressure, temperature, ion species (H_3O^+, protonated pentanal, protonated 2,6-DTBP), or the collision model (HSS vs. GR-HSS), but follow the same structure.

Furthermore, the associated dataset contains two reproducibility workflows. The first matched σ_{event} workflow runs three validation cases for 300 K and 20 Pa. In the first one, HSS uses the specified HSS value of σ_{event} for Poisson sampling, while GR-HSS uses the stored orientation-resolved values of σ_{event} (as described in Section S3), which serves as the baseline simulation. In the second run, GR-HSS keeps its relative orientations between ion and neutral and its geometry-resolved scattering, but all values of σ_{event} are set to the fixed HSS value. In the third run, HSS uses the mean GR-HSS value of σ_{event}, while GR-HSS uses its usual distribution of orientation-resolved values.

This workflow should answer whether differences in equilibration time between the HSS and GR-HSS models can be attributed to different collision rates arising from different values of σ_{event} used in Poisson sampling. If that is the case, the ratio of the equilibration times is expected to approach unity within statistical uncertainty.

In a second additional workflow, ten runs with independent RNG seed values were performed at three representative temperature and pressure conditions for each ion to quantify the statistical uncertainty of the thermalization case and the robustness of the fitted equilibration time, as described in the Section S5.

Analysis

The instantaneous translational temperature of the ion ensemble is calculated directly from the velocity data stored in the HDF5 trajectory output files. For each timestep, the translational temperature is obtained from the mean kinetic energy according to $T_{\text{ens}} = \langle m_{\text{ion}} v^2 \rangle / 3k_{\text{B}}$.

Speed distributions are evaluated from the speed magnitudes $|v|$ of the ions in the final 10 time steps of the trajectory. These speeds are binned into normalized density histograms and compared with analytical Maxwell distributions.

Thermalization times are determined from the time evolution of the translational temperature. The primary estimate is based on an exponential relaxation model according to eq. (S31), where T_{target} is the domain temperature and T_0 is the initial translational temperature.

$$T(t) = T_{\text{target}} - \left(T_{\text{target}} - T_0\right) \exp\left(-\frac{t}{\tau}\right) \quad \text{(S31)}$$

The fit is performed in linearized form using the variable $f = (T - T_0) / (T_{\text{target}} - T_0)$, where f describes the fraction of the temperature difference between T_{target} and T_0, leading to $\ln(1-f) = a - t/\tau$. By default, only the intermediate progress range from 10% to 90% of the domain temperature is included in the fit to reduce sensitivity to the noisy asymptotic tail. The fitted relaxation constant τ is then used to calculate the 95% and 99% thermalization times as $-\ln(0.05)\tau$ and $-\ln(0.01)\tau$, respectively.

For comparison, simple threshold-based thermalization times are also exported. These are defined as the first timestep at which the translational temperature exceeds 95% or 99% of the domain temperature. In addition, a tolerance-window estimate is included, which identifies the first timestep from which the translational temperature remains within a specified relative tolerance of the domain temperature for a fixed number of consecutive sampled points. These threshold-based quantities are more sensitive to statistical noise and to the selected timestep spacing than the fit-based relaxation times.

The same postprocessing procedure is applied to the output files generated by the full-grid analysis, the matched σ_{event} reproducibility workflow, and the statistical uncertainty workflow. The resulting analysis files in the associated dataset include temperature curves, speed distributions, Maxwell reference distributions, fit-based relaxation times, and threshold-based comparison metrics.

Reaction Kinetics Case

Configuration

The bimolecular reaction kinetics validation illustrates the use of stochastic reactions in ICARION. The example file *proton_transfer_h3o_pentanal_k1.json* in the associated dataset simulates a proton transfer between H_3O^+ and pentanal at 400 Pa and 300 K. This case demonstrates reaction handling in the presence of a non-reactive background gas containing trace amounts of a reactive species.

Key settings:

- **Simulation parameters**: As in the thermalization case, a time step sufficiently small to make multiple reaction events within one time step negligible is used (100 ns).
- **Reaction model**: Reactions are enabled (`enable_reactions = true`). The system contains two neutral components:
    - Helium, which participates in collisions but not in reactions.
    - Pentanal, present at 1 ppm, which participates only in reactions.
- **Ion initialization**: H_3O^+ ions are initialized thermally and propagate under field-free conditions.
- **Reaction database**: The reaction is defined in *h3o_pentanal_k1_reactions.json,* provided in the associated dataset, as a bimolecular proton transfer with a rate coefficient of $1 \cdot 10^{-15}$ m^3/s ($1 \cdot 10^{-9}$ cm^3/s), producing a protonated pentanal ion.

This example demonstrates that ICARION accurately reproduces exponential decay behavior under pseudo-first-order conditions, as shown in the main manuscript. Variants of this configuration explore different rate coefficients ($2 \cdot 10^{-9}$ cm^3/s and $3 \cdot 10^{-9}$ cm^3/s) to assess stochastic consistency.

Analysis

For each stored trajectory time point, reagent and product ion counts are extracted directly from the HDF5 output files and compared to the analytical pseudo-first-order reaction solution. The pseudo-first-order rate constant is calculated from the bimolecular rate constant and the neutral density, $k_{1,\text{eff}} = k_1 N_{\text{neutral}}$, with $N_{\text{neutral}} = x_{\text{neutral}}\, p/k_{\text{B}}T$ obtained from the simulation configuration. The analytical curves are given by $N_{\text{reagent}}(t)$

$= N_0 \exp(-k_{1,\mathrm{eff}}\, t)$ and $N_{\mathrm{product}}(t) = N_0 - N_{\mathrm{reagent}}(t)$, where N_0 is the initial total number of ions.

Ion Mobility Configuration

The ion mobility benchmark compares ICARION against SIMION and IDSimF in a uniform-field drift cell operated at various E/N values and helium pressures. Simulations were evaluated based on the obtained reduced ion mobility K_0 using identical physical and geometric parameters across all tools.

Common Evaluation Method

In all cases, the reduced ion mobility was determined from the drift time t_{D} according to eq. (S32), where L_{eff} is the effective drift length measured between ion injection and detection, N_0 the Loschmidt constant, and E/N the reduced electric field strength. Arrival times were fitted with a Gaussian distribution to extract t_{D}. All simulations varied pressure (20-2000 Pa) and E/N (3, 5, 7, 8.5, and 10 Td)

$$K_0 = \frac{L_{\mathrm{eff}}}{t_{\mathrm{D}} N_0 E/N} \tag{S32}$$

ICARION Configuration

The example file *ims_ehss_PentanalHp_2000Pa_10Td_comments.json* in the associated dataset defines the ICARION simulation parameters.

Key settings:

- **Integrator and time step**: RK4 scheme with the time step set to 1/50 of the mean collision time.
- **Ion injection**: 1,000 protonated pentanal ions are initialized with a thermal velocity distribution at 300 K and centered at (0, 0, 0) with a Gaussian standard deviation of 0.5 mm.
- **Domain**: The axial origin is set to -10 mm, with a domain length of 110 mm, yielding an effective drift length of 100 mm.
- **Electric field**: Specified via the reduced field (E/N = 10 Td), with the electric field calculated at runtime from pressure and temperature.

Other configurations vary in E/N, pressure, or collision model (HSS vs. GR-HSS). For the cross-code isotropic single-sphere comparison between ICARION-HSS, IDSimF, and SIMION, the same effective CCS value of 53.9 Å^2 was used. GR-HSS instead uses the orientation-dependent projected areas described in Section S3.

Similar to the thermalization statistical uncertainty workflow, the ion mobility case includes an additional workflow, which conducts five runs with different RNG seed values for the same condition (300 K, 20 Pa, and 10 Td, with the remaining settings as in the full-grid runs described above) to validate the statistical uncertainty of mobility analysis with this workflow for a given set of conditions.

SIMION Configuration

A 102 mm drift cell was constructed from 20 equidistant ring electrodes (5 mm center spacing, 3 mm width), with entrance and exit end caps to reduce boundary effects (see Figure S2). The field was defined by assigning linearly scaled potentials to the rings relative to the grounded exit end cap. Geometry and exemplary run configuration files are provided in the associated dataset.

Key details:

- **Ion injection**: 1,000 protonated pentanal ions are created at x = 4 mm and radially centered with a directional cone (half-angle 45°) to prevent immediate ion loss at the entrance after generation.
- **Initial velocity**: Velocities are sampled from a Gaussian distribution with a mean energy of 0.039 eV and a standard deviation of 0.032 eV, approximating a thermal distribution at 300 K.
- **Drift length**: Effective drift length L_{eff} = 98 mm (from x = 4 mm to x = 102 mm).
- **Collision model**: HS1 model with a CCS value of 53.9 Å^2 at an ambient temperature of 300 K.

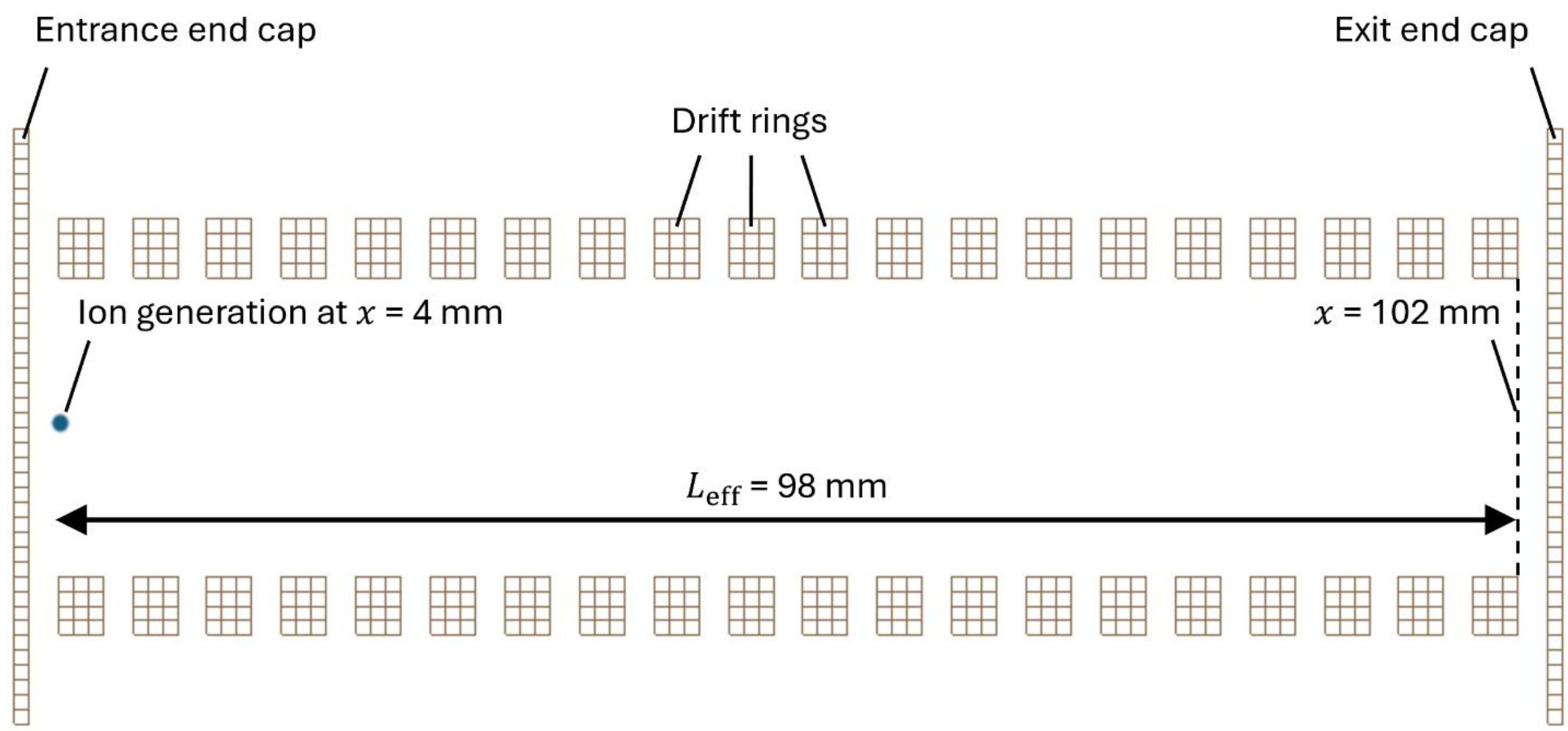

Figure S2. Schematic layout of the drift cell used in the SIMION simulations. Ions are generated near the entrance end cap at $x = 4\,mm$ and traverse a sequence of equally spaced drift rings toward the exit end cap at $x = 102\,mm$. The effective drift length $L_{eff} = 98\,mm$ is defined as the distance between the ion creation position and the exit plane. End caps provide field termination, while the ring stack establishes a nominally uniform electric field along the drift axis.

IDSimF Configuration

All simulations used the *IMSSim* example application from the IDSimF repository, with a 100 mm drift cell and a uniform electric field.

Key settings:

- **Simulation**: The time step is set to 1/50 of the mean collision time at the respective pressure and temperature.
- **Ion injection**: 1,000 protonated pentanal ions are created per run in a 1 mm box (x/y/z) centered at the origin.
- **Collision model**: Isotropic single-sphere scattering model (*btree_HS*). Since this model is parameterized using the kinetic diameters of the ion and neutral rather than by a directly specified CCS, the diameters were chosen to yield a cross section of 53.9 Å^2.
- **Electric field**: Explicitly set to match the reduced electric field values used in ICARION simulations.

Preprocessing scripts and all configuration files are available in the associated dataset.

Multi-Domain Case

Configuration

The file *drift_quad_coupling_comments.json* in the associated dataset demonstrates the coupling of two distinct domains within a single ICARION simulation: a drift cell and a quadrupole mass filter, arranged sequentially along the global z-axis.

Key settings:

- **Domain 1: IMS**
  - Geometry: Cylindrical geometry with a length of 100 mm and a radius of 10 mm.
  - Environment: The environment is set to 298 K and 1000 Pa (helium).
  - Electric field: Uniform axial electric field calculated at runtime from the specified E/N of 20 Td.
- **Domain 2: Quadrupole**
  - Geometry: Cylindrical geometry with a length of 100 mm and a radius of 4 mm.
  - Environment: The environment is set to 298 K and $1 \cdot 10^{-4}$ Pa (helium).

- Electric field: To each electrode, an RF voltage of 400 V (0-peak) at a frequency of 2 MHz is applied, with a 180° phase difference between adjacent rods. Between the two pairs of electrodes, an additional DC voltage of 50 V was applied. The uniform axial electric field is calculated from the specified axial voltage of 10 V and the domain length of 100 mm.

This example illustrates ICARION's ability to simulate multi-domain systems with heterogeneous field configurations and pressure regimes. In the three simulations, the applied RF voltage as well as the DC voltage between the electrode pair were varied to set the quadrupole mass filter to transmission for only one of the three ion species, enabling selective m/z filtering. These operating points were set near the high-q (0.642-0.732) apex region of the Mathieu stability diagram, but deliberately below the apex in the a-parameter (between 0.161 and 0.176), providing m/z-selective transmission of the target ion species while shifting the non-targeted ions outside the first stability region.

Analysis

For the multi-domain analysis, arrival times at the IMS-quadrupole interface were extracted directly from the HDF5 trajectory output files. For each ion, the arrival time was defined as the first time point in the stored trajectory data at which the ion entered the quadrupole domain, corresponding to transfer from the IMS domain into the quadrupole domain. The resulting per-ion arrival times were exported together with the corresponding species labels and used to construct arrival-time distributions.

Quadrupole transmission ratios were determined from the axial positions reached within the quadrupole domain. For each ion, the maximum axial position sampled while the ion was assigned to the quadrupole domain was compared to the axial end position of the quadrupole. Ions reaching this end position within the applied numerical tolerance were counted as transmitted. Transmission fractions were calculated separately for each species as the number of transmitted ions divided by the total number of launched ions of that species.

Performance Benchmark

Configuration

The file *ims_basic_N1000.json* in the associated dataset illustrates the file structure used for benchmarking ICARION's computational performance as a function of ion count and CPU thread count. The configuration is based on a simple drift cell setup and does not focus on ion mobility transport, but on performance benchmarking. Comparison benchmarks were performed with IDSimF and SIMION using the same setups described above. In those configurations, the environmental and instrumental parameters were matched to the ICARION simulations, and the comparable single hard-sphere collision

models were used. In SIMION, the ions were initialized at x = 82 mm to yield the same drift length of 20 mm. SIMION was run in single-thread mode using adaptive integration with *T.Qual*=3 and a maximum time step of 0.6 ns.

Key settings:

- **Ion species**: protonated pentanal
- **Integrator**: RK4 with a time step of 0.6 ns to ensure that it is small enough to make multiple collision events within one time step negligible.
- **Geometry**: Cylindrical domain with an effective drift length of 20 mm
- **Environment**: The environment is set to 300 K and 200 Pa (helium).
- **Electric field**: Calculated at runtime from the specified E/N of 10 Td.
- **Collision model**: Isotropic hard-sphere (HSS) model.

Benchmark parameters:

- Ion counts: 1,000 / 5,000 / 10,000 / 50,000
- Threads: 1 / 2 / 4 / 8 / 16 (OpenMP parallelization)
- Each benchmark condition includes five simulation runs plus one warm-up run to reduce initialization effects.

These runs are intended to measure wall-clock time and parallel speedup on shared-memory CPU systems. Absolute ion mobility accuracy is not the focus of this benchmark and is evaluated in the transport validation cases above. Raw timing results are available in the associated dataset, while the corresponding scaling plots are shown in the main manuscript.

Analysis

For the benchmark analysis, runtime performance and peak memory usage metrics were evaluated. The wall-clock time and maximum resident set size (RSS) were measured externally by the benchmark runner for each ICARION execution and written to the raw benchmark CSV files. The postprocessing step aggregates these per-run wall-clock times across repeated runs for each combination of ion number and thread count, reporting the median, mean, and standard deviation, as well as thread-scaling speedup, parallel efficiency, and maximum RSS.

S5. Additional Thermalization Data

This section summarizes the main findings of the control simulations (I) that use additional RNG seed values to validate statistical uncertainty and (II) that set σ_{event} to the same value to disentangle the effects of collision rate and momentum transfer in the GR-HSS and HSS models. The raw data and tables containing the detailed analysis outputs are summarized in the associated dataset.

The results of the control simulations are summarized in Tables S2 and S3. In the workflow for validating statistical uncertainty, ten independent RNG seed values were evaluated at three representative sets of temperature and pressure for each ion. The fitted $\tau_{\mathrm{HSS}}/\tau_{\mathrm{GR-HSS}}$ remains in the same range as predicted by σ_{event}, with standard deviations of approximately 0.01 – 0.04 across seed values for a given ion and fixed condition. The variation between different conditions is of similar magnitude and is attributed to stochastic sampling and fit uncertainty rather than to a pressure- or temperature-dependent effect.

As a mechanistic control, another workflow, which uses the same σ_{event} value for both models was performed for protonated 2,6-DTBP. In a first simulation, GR-HSS was run with its geometry-resolved scattering kept, but with all values of σ_{event} set to the corresponding HSS value so that the expected collision rates match. In a second simulation, GR-HSS keeps both its scattering and the distribution of σ_{event} values and the value of σ_{event} in the HSS simulation is set to the mean GR-HSS value. All runs are repeated with five independent RNG seed values. Under these matched conditions, the large offset in equilibration times nearly disappeared: the fitted ratio decreased from about 1.210 in the baseline simulation to 1.043 ± 0.007 or 1.019 ± 0.015 in the two control simulations. This strongly suggests that the dominant contribution comes from the higher collision rate in the GR-HSS model in the baseline simulation. Therefore, additional differences resulting from the differences in geometry-resolved and isotropic scattering seem to be comparatively small, on the order of a few percent in the investigated validation cases, but not entirely absent.

Table S2. Collision event cross section σ_{event} ratios and thermalization time ratios from RNG seed variation control simulation. All simulations were run with ten different RNG seed values. The mean values of σ_{event} in GR-HSS are 30.77, 66.96, and 99.95 Å^2 for H_3O^+, protonated pentanal, and protonated 2,6-DTBP. The values of σ_{event} in HSS are 24.7, 53.9, and 85.0 Å^2 for H_3O^+, protonated pentanal, and protonated 2,6-DTBP.

Ion	T [K]	p [Pa]	$\tau_{\mathrm{HSS}}/\tau_{\mathrm{GR-HSS}}$	σ_{event} ratio (mean) GR-HSS / HSS
H_3O^+	300	20	1.279 ± 0.013	1.236
H_3O^+	300	200	1.176 ± 0.041	1.236
H_3O^+	700	200	1.204 ± 0.013	1.236
protonated pentanal	300	20	1.206 ± 0.019	1.247
protonated pentanal	300	200	1.244 ± 0.025	1.247
protonated pentanal	700	200	1.251 ± 0.009	1.247
protonated 2,6-DTBP	300	20	1.167 ± 0.027	1.165
protonated 2,6-DTBP	300	200	1.197 ± 0.031	1.165
protonated 2,6-DTBP	700	200	1.152 ± 0.026	1.165

Table S3. Results of matched σ_{event} control simulations for protonated 2,6-DTBP. All simulations used five independent RNG seed values. The mean value of σ_{event} in GR-HSS is 99.95 Å^2, and the corresponding value in HSS is 85.0 Å^2, leading to a nominal ratio of 1.165.

Control mode	Description	$\tau_{\mathrm{HSS}}/\tau_{\mathrm{GR-HSS}}$
Baseline	HSS uses MobCal-derived value; GR-HSS uses orientation-resolved σ_{event}	1.210 ± 0.014
GR-HSS with HSS σ_{event}	GR-HSS keeps geometry-resolved scattering, but uses σ_{event} from HSS	1.043 ± 0.007
HSS with mean GR-HSS σ_{event}	HSS uses mean σ_{event} from GR-HSS; GR-HSS uses orientation-resolved σ_{event}	1.019 ± 0.015

S6. Additional Ion Mobility Data

To evaluate the sensitivity of simulated ion mobility to the initial velocity distribution of injected ions, four distinct initial conditions were tested for protonated pentanal in helium using the HSS model: directed injection with 1.0 eV or 0.5 eV axial kinetic energy and thermalized isotropic distributions at 0.1 K or 1000 K. Figure S3 shows the relative deviation in apparent reduced ion mobility for these four cases relative to the reduced ion mobility obtained using an initial thermal distribution at 300 K, evaluated for different pressures and E/N values.

At the highest pressure of 500 Pa, all cases nearly converge to the same reduced ion mobility, confirming rapid collisional equilibration and reduced sensitivity to initial conditions. In contrast, at low pressures, the reduced number of collisions leads to slower equilibration within the drift region. As a result, the apparent reduced ion mobility retains a memory of the initial velocity distribution, producing systematic deviations of up to 8 % depending on the injection energy and directionality.

These deviations are largely independent of E/N, indicating that the pressure-dependent collision frequency, rather than the reduced electric field strength, is the dominant factor causing the observed deviations.

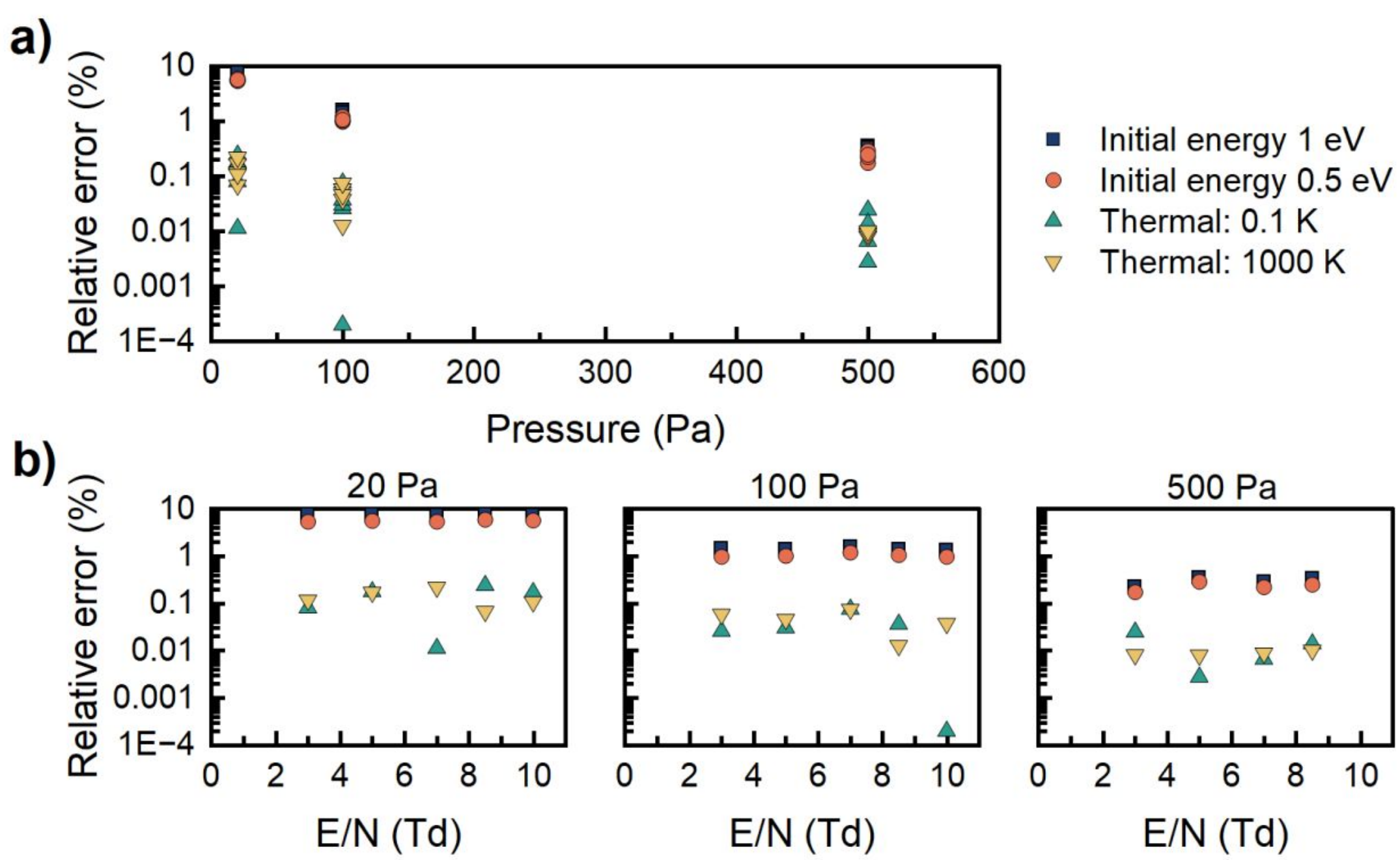

Figure S3. Relative ion mobility error for protonated pentanal in helium at 300 K, referenced to the apparent reduced ion mobility obtained using an initial thermalized distribution at 300 K, as a function of a) pressure (for different E/N values of 3, 5, 7, 8.5, and 10 Td) and b) reduced electric field strength (E/N) for pressures of 20 Pa (left), 100 Pa (center), and 500 Pa (right). The simulations are conducted for four different initial velocity distributions: directed injections at 1.0 eV and 0.5 eV (squares and circles) and isotropic thermal distributions at 0.1 K and 1000 K (triangles) using the HSS model.

Again, we conducted control simulations to validate the effects of different RNG seed values and matched σ_{event} values.

In the statistical uncertainty simulation, 10 independent RNG seed values are evaluated at three representative IMS conditions for protonated pentanal. The resulting ratios in reduced ion mobility between HSS and GR-HSS were 1.258 ± 0.001 at 200 Pa and 5 Td, 1.256 ± 0.003 at 200 Pa and 10 Td, and 1.256 ± 0.001 at 500 Pa and 10 Td. These values closely match the ratio of σ_{event} of 1.247.

In the control simulation using the same σ_{event} value, GR-HSS was run with geometry-resolved scattering kept, but with all values of σ_{event} set to the fixed HSS value obtained by MobCal-MPI (see main manuscript). Under these conditions, the ion mobility offset nearly disappeared, with the obtained ion mobility ratio decreasing to 1.007 ± 0.001. Conversely, when HSS was run with the mean σ_{event} GR-HSS value, the ion mobility ratio was 1.014 ± 0.003. These control simulations suggest that the ion mobility difference is dominated by different collision rates, with only a small residual difference arising from the different collision physics.

References

(1) Schaefer, C.; Jašík, J.; Španěl, P. *Data for 'ICARION: A Modular Framework for Ion Trajectory Simulation in Electric Fields and Neutral Gas Environments'*, National Data Repository, 2026. https://doi.org/10.48700/6t3q1-es569.
(2) Haack, A.; Ieritano, C.; Hopkins, W. S. MobCal-MPI 2.0: an accurate and parallelized package for calculating field-dependent collision cross sections and ion mobilities. *Analyst* **2023**, *148* (14), 3257–3273. DOI: 10.1039/d3an00545c.
(3) Robertson, S.; Sternovsky, Z. Monte Carlo model of ion mobility and diffusion for low and high electric fields. *Phys. Rev. E* **2003**, *67* (4), 046405. DOI: 10.1103/PhysRevE.67.046405.
(4) Mantina, M.; Chamberlin, A. C.; Valero, R.; Cramer, C. J.; Truhlar, D. G. Consistent van der Waals radii for the whole main group. *J. Phys. Chem. A* **2009**, *113* (19), 5806–5812. DOI: 10.1021/jp8111556
(5) Dryahina, K.; Španěl, P. A convenient method for calculation of ionic diffusion coefficients for accurate selected ion flow tube mass spectrometry, SIFT-MS. *Int. J. Mass Spectrom.* **2005**, *244* (2-3), 148–154. DOI: 10.1016/j.ijms.2005.06.001.
(6) Mesleh, M. F.; Hunter, J. M.; Shvartsburg, A. A.; Schatz, G. C.; Jarrold, M. F. Structural Information from Ion Mobility Measurements: Effects of the Long-Range Potential. *J. Phys. Chem.* **1996**, *100* (40), 16082–16086. DOI: 10.1021/jp961623v.
(7) Bleiholder, C.; Wyttenbach, T.; Bowers, M. T. A novel projection approximation algorithm for the fast and accurate computation of molecular collision cross sections (I). Method. *Int. J. Mass Spectrom.* **2011**, *308* (1), 1–10. DOI: 10.1016/j.ijms.2011.06.014.
(8) Shvartsburg, A. A.; Jarrold, M. F. An exact hard-spheres scattering model for the mobilities of polyatomic ions. *Chem. Phys. Lett.* **1996**, *261* (1-2), 86–91. DOI: 10.1016/0009-2614(96)00941-4.

(9) Larriba-Andaluz, C.; Hogan, C. J. Ion mobilities in diatomic gases: measurement versus prediction with non-specular scattering models. *J. Phys. Chem. A* **2013**, *117* (19), 3887–3901. DOI: 10.1021/jp312432z.
(10) Larriba-Andaluz, C.; Hogan, C. J. Free molecular collision cross section calculation methods for nanoparticles and complex ions with energy accommodation. *J. Comput. Phys.* **2013**, *251*, 344–363. DOI: 10.1016/j.jcp.2013.05.038.
(11) Epstein, P. S. On the Resistance Experienced by Spheres in their Motion through Gases. *Phys. Rev.* **1924**, *23* (6), 710–733. DOI: 10.1103/PhysRev.23.710.
(12) Schaefer, C. *ICARION: Ion Collision And Reaction IntegratiON*; Zenodo, 2026. DOI: 10.5281/zenodo.20455416.
